# DDX5 deficiency drives non-canonical NF-κB activation and NRF2 expression, influencing sorafenib response and hepatocellular carcinoma progression

**DOI:** 10.1038/s41419-024-06977-z

**Published:** 2024-08-09

**Authors:** Zhili Li, Woojun Kim, Sagar Utturkar, Bingyu Yan, Nadia Atallah Lanman, Bennett D. Elzey, Majid Kazemian, Yoon Yeo, Ourania Andrisani

**Affiliations:** 1 Department of Basic Medical Sciences, Purdue University, West Lafayette, IN USA; 2 Purdue Institute for Cancer Research, West Lafayette, IN USA; 3 Department of Industrial and Physical Pharmacy, Purdue University, West Lafayette, IN USA; 4 Department of Biochemistry, Purdue University, West Lafayette, IN USA; 5 Department of Comparative Pathobiology, Purdue University, West Lafayette, IN USA; 6 Department of Computer Science, Purdue University, West Lafayette, IN USA

**Keywords:** Cancer, Molecular biology

## Abstract

In advanced hepatocellular carcinoma (HCC), RNA helicase DDX5 regulates the Wnt/β-catenin-ferroptosis axis, influencing the efficacy of the multi-tyrosine kinase inhibitor (mTKI) sorafenib. DDX5 inhibits Wnt/β-catenin signaling, preventing sorafenib-induced ferroptosis escape. Sorafenib/mTKIs reduce DDX5 expression, correlating with poor patient survival post-sorafenib treatment. Notably, DDX5-knockout in HCC cells activates Wnt/β-catenin signaling persistently. Herein, we investigate the mechanistic impact of Wnt/β-catenin activation resulting from DDX5 downregulation in the progression and treatment of HCC. RNAseq analyses identified shared genes repressed by DDX5 and upregulated by sorafenib, including Wnt signaling genes, *NF-κB-inducing kinase* (*NIK)* essential for non-canonical NF-κB (p52/RelB) activation, and cytoprotective transcription factor *NRF2*. We demonstrate, Wnt/β-catenin activation induced *NIK* transcription, leading to non-canonical NF-κB activation, which subsequently mediated *NRF2* transcription. Additionally, DDX5 deficiency extended NRF2 protein half-life by inactivating KEAP1 through p62/SQSTM1 stabilization. In a preclinical HCC mouse model, *NRF2* knockdown or DDX5 overexpression restricted tumor growth upon sorafenib treatment, via induction of ferroptosis. Importantly, DDX5-knockout HCC cells exhibited elevated expression of Wnt signaling genes, *NIK, p52/RelB*, and NRF2-regulated genes, regardless of sorafenib treatment. Transcriptomic analyses of HCCs from TCGA and the Stelic Animal Model (STAM) of non-alcoholic steatohepatitis revealed elevated expression of these interconnected pathways in the context of DDX5 downregulation. In conclusion, DDX5 deficiency triggers Wnt/β-catenin signaling, promoting p52/RelB and NRF2 activation, thereby enabling ferroptosis evasion upon sorafenib treatment. Similarly, independent of sorafenib, DDX5 deficiency in liver tumors enhances activation and gene expression of these interconnected pathways, underscoring the clinical relevance of DDX5 deficiency in HCC progression and therapeutic response.

## Introduction

Hepatocellular carcinoma (HCC) is a leading primary cancer with increasing global incidence [1]. Standard treatments for early-stage HCC include surgical resection, liver transplantation, and percutaneous ablation. In advanced HCC, combination therapy targeting both VEGF (bevacizumab) and PD-L1 (atezolizumab) is the primary choice [2]. However, multi-tyrosine kinase inhibitors (mTKIs) including sorafenib are still widely used when immunotherapy is contra-indicated or fails [3]. Since sorafenib/mTKIs are still the gold standard in second or later-line systemic therapy, determining the mechanisms of mTKI sensitivity becomes imperative for enhancing antitumor efficacy through innovative therapeutic strategies.

Previous studies, on the mechanism of sorafenib resistance in HCC [4, 5], have linked ferroptosis escape to activation of the cytoprotective transcription factor NRF2 [6], through an unknown mechanism. Our recent studies have established a mechanistic link between sorafenib antitumor efficacy and the expression level of the RNA helicase DDX5 [7]. DDX5 regulates various RNA-related processes in vivo [8]. In HCC cell lines, DDX5 interacts with the epigenetic silencing PRC2 complex [9], controls STAT1 translation by resolving a G-quadruplex located at the 5’UTR of the STAT1 mRNA [10], and its knockdown (DDX5^KD^) leads to hepatosphere formation [11]. Notably, preclinical and clinical evidence indicates that DDX5 deficiency in sorafenib-treated HCC cells orchestrates activation of signaling cascades enabling ferroptosis escape [7], a mechanism linked to drug resistance [12, 13]. Specifically, DDX5 deficiency enables enhanced expression of DVL1, a key regulator of Wnt signaling activation [14], a poor prognostic indicator of disease progression [15], and sorafenib response [7]. Conversely, inhibition of Wnt signaling or ectopic expression of DDX5 enhances the antitumor efficacy of sorafenib through ferroptosis [7].

Ferroptosis, a non-apoptotic form of regulated cell death [16], is characterized by membrane lipid peroxidation induced by ferrous iron (Fe^2+)^ under conditions of increased reactive oxygen species (ROS) [16]. In HCC cell lines treated with ferroptosis-inducing agents, increased levels of p62/SQSTM1 promote KEAP1 inactivation and subsequent NRF2 stabilization [6]. In turn, NRF2 mediates expression of genes involved in glutathione production and ROS detoxification [17]. Intriguingly, KEAP1 has been identified as a sorafenib/mTKI sensitivity gene in HCC [18], yet the mechanism underlying KEAP1 loss in sorafenib resistance remains elusive. Prior studies by others have demonstrated the crucial roles of both NRF2 [19] and p62/SQSTM1 [20] in the pathogenesis of HCC. Interestingly, the stability of p62/SQSTM1, a key player in KEAP1 inactivation, is regulated by DDX5 [21]. Based on these findings, we hypothesize that DDX5 orchestrates additional events linked to ferroptosis and sorafenib sensitivity.

Since DDX5 knockout in HCC cells leads to persistent activation of Wnt/β-catenin signaling [7], herein, our investigations focused on the impact of Wnt/β-catenin activation in HCC progression and treatment outcomes. We provide evidence that Wnt/β-catenin signaling transcriptionally induced the expression of *NF-κB-inducing kinase (NIK*), encoded by the *MAP3K14* gene. NIK expression is the hallmark of non-canonical (p52/RelB) NF-κB activation [22], a pathway associated with the stem cell phenotype in various cancers, including lung [23] and breast [24] cancers. In hepatocytes, NIK orchestrates the progression from non-alcoholic steatohepatitis (NASH) to HCC [25]. Interestingly, this NASH to HCC transition involves the transcriptional downregulation of DDX5, and studies have shown that restoring DDX5 alleviates NASH progression [26]. Furthermore, recent studies have established an intriguing connection between the expression and nuclear localization of RelB, a key subunit of non-canonical NF-κB, and increased aggressiveness and poor prognosis of HCC [27].

In this study, we present evidence indicating that the reduction of DDX5 by sorafenib activated the non-canonical NF-κB pathway via Wnt/β-catenin signaling. Subsequently, this activation of non-canonical NF-κB mediated increased expression of the transcription factor *NRF2*. In addition, the absence of DDX5 was found to be associated with prolonged NRF2 protein stability, mediated by p62/SQSTM1[21], which targets KEAP1 inactivation. Moreover, we demonstrate that DDX5 knockout HCC cells display sustained activation of Wnt signaling, as well as of the interconnected non-canonical NF-κB and NRF2 pathways, even in the absence of sorafenib treatment. This observation prompted examination of transcriptomic datasets featuring DDX5 downregulation, including those from the STAM NASH to HCC model [28, 29] and human HCCs from TCGA. Bioinformatic analyses revealed a connection between DDX5 deficiency and the activation of the interlinked Wnt/β-catenin-non-canonical NF-κB-NRF2 pathways both during the NASH to HCC progression and in advanced human HCCs.

## Materials and methods

### Cell culture

Human HCC cell lines used include WT HepAD38 [30], DDX5 knockdown (DDX5^KD^) HepAD38 [11], Dox-inducible HepaRG-FLAG-DDX5 [10], Dox-inducible Huh7-DDX5, and HepAD38-DDX5, and Huh7-DDX5 knockout (DDX5^KO^) grown as described [7, 10]. The cell lines were routinely tested for mycoplasma. HepAD38 cell lines were authenticated by short tandem repeat (STR) analysis performed by ATCC.

### Transfection assays

HCC cell lines (5 × 10^4^ cells) were transfected with 100 ng of indicated vectors, including ToPFlash (TCF/LEF-Firefly Luciferase), NF-KB-Luciferase, or *MAP3K14*-Luciferase [31] and co-transfected with Renilla luciferase (100 ng). The siRNAs (50 pM) were transfected using RNAiMax (Life Technologies). Luciferase activity measured 48 h after transfection using the Dual Luciferase Assay System, according to manufacturer’s protocol (Promega), and normalized to Renilla luciferase. The plasmids and siRNAs used are listed in Supplementary Table S1.

### Cell viability assays

HCC cells (1 × 10^4^) seeded in 96-well plates were treated with DMSO, sorafenib (7.5 μM), B022 (5.0 µM), XAV939 (20 µM), or transfected with 50 pM siRNAs for 24 h. Growth inhibition was measured at 490 nm using the CellTiter 96 AQ_ueous_ One Solution Cell Proliferation assay (Promega). Viability (100%) refers to A_490_ value of DMSO-treated cells. The background absorbance was measured in wells containing medium and MTS without cells.

### Mice

Severely immunocompromised NRG mice (non-obese diabetic rag knock out IL-2 common γ chain knock out) were obtained from our breeding colony (The Jackson Laboratory stock # 007799) and were maintained *ad libitum* on standard chow diet (Teklad 2918) and water acidified to pH 2.5–3.0 with acetic acid. Mice were used in experiments at ages 6-12 weeks.

### Huh7 xenografts

Tumor xenografts were established by subcutaneous injection of 5 × 10^6^ Huh7 cells per NRG mouse. When tumors reached mean volume of ~70–100 mm^3^, mice were assigned to control and treated groups to achieve closely matched mean tumor volume and standard deviation and received vehicle (5% DMSO + 45% PEG400) or sorafenib orally at 40 mg/kg daily for first 7 days, followed by 80 mg/kg daily for remaining two weeks. Huh7 DDX5 overexpressing tumor-bearing mice were generated using Dox-inducible Huh7-DDX5 cells. Doxycycline (Dox)-containing H_2_O (1.0 µg/ml) was fed to half of the mice, 48 h prior to daily administration of sorafenib (80 mg/kg, 5days/week), when tumor volume reached 50–70 mm^3^.

### Nanosac preparation

Nanosacs carrying siCtrl or siNRF2 were prepared as previously described [32]. Nanosac-encapsulated siRNAs were administered every 48 h intra-tumorally, delivering 3.0 µg siRNA per injection. The detailed protocol for Nanosac preparation is described in [7].

**Immunoblotting** was performed as described in the Supplementary Material. Antibodies used are listed in Supplementary Table S2.

### RNA preparation and qRT-PCR

Methods are described in the Supplementary Information section; primer sequences are listed in Supplementary Table S3, and reagents, chemical inhibitors, and kits are listed in Supplementary Table S4.

### RNA-seq analysis

Detailed methods of transcriptomic analyses of WT HepAD38 [30] and DDX5^KD^ cells [11] treated with sorafenib are described in [7]. Gene set enrichment analysis (GSEA) was performed using GSEA software [33].

### Statistical analysis

Statistical analysis was performed using the unpaired *t* test in GraphPad Prism (version 6.0; GraphPad Software, San Diego, CA, USA). Differences were considered statistically significant at *P* < 0.05.

## Results

### DDX5 downregulation promotes expression of non-canonical NF-κB signaling genes

**Fig. 1:**
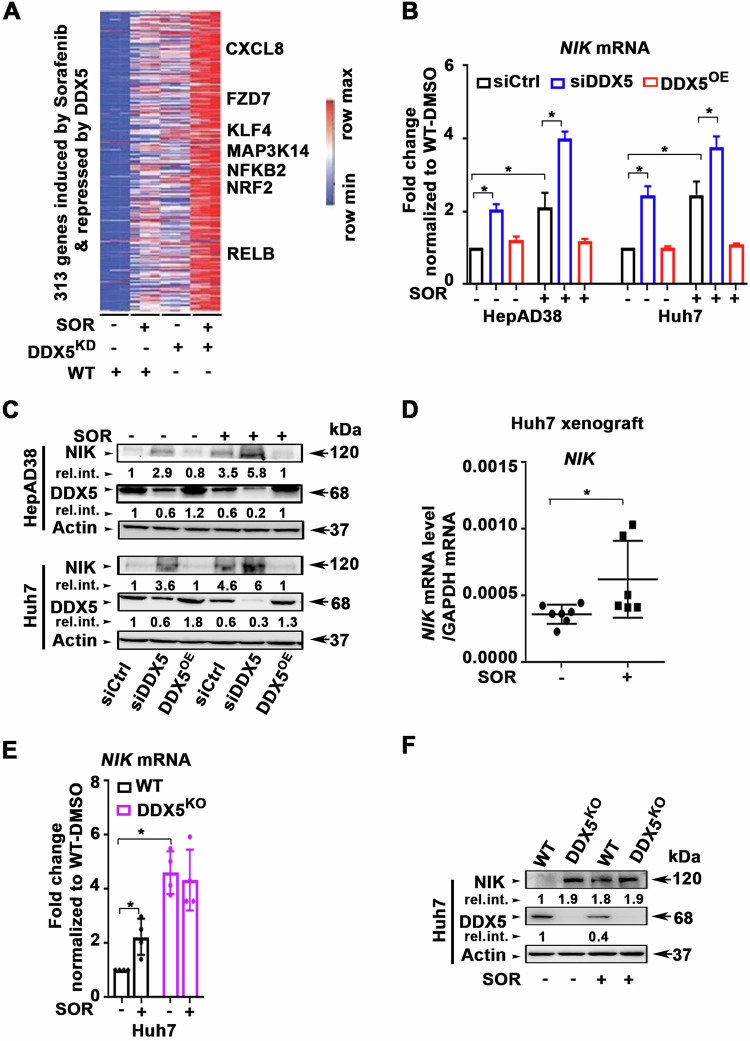
Sorafenib-induced and DDX5-repressed genes enriched in Wnt/β-catenin and non-canonical NF-κB signaling. **A** Heatmap of common genes between SOR-induced and DDX5-repressed genes, as described in [7]. **B** RT-PCR quantification of *NIK* mRNA using total RNA from HepAD38 and Huh7 cells transfected with control (siCtrl) or DDX5 siRNAs. DDX5 overexpression (DDX5^OE^) using Dox-inducible-DDX5 cell lines grown with Dox (1.0 µg/ml) for 48 h and SOR for the last 24 h. **C** Immunoblots of NIK using lysates from HepAD38 and Huh7 cell lines transfected with siCtrl or siDDX5 ±10 µM SOR for 24 h. For DDX5^OE^, Dox-inducible-DDX5 cell lines grown with Dox (1.0 µg/ml) for 48 h and SOR for the last 24 h. A representative immunoblot from three independent experiments (n = 3). Quantification shown in Supplementary Fig. S1. **D** RT-PCR quantification of *NIK* mRNA using total RNA isolated from Huh7 xenograft tumors, treated ± SOR, as indicated. Data expressed as mean ± SEM from eight tumors, described in Li et al. [7]. *p < 0.05 by unpaired *t* test. **E**, **F** qRT-PCR of *NIK* mRNA and immunoblots of NIK protein, using total RNA or lysates, respectively, isolated from WT and DDX5^KO^ Huh7 cells, ±SOR (10 µM) for 24 h, as indicated. **E** qRT-PCR data expressed as mean ± SEM from n = 3. *p < 0.05, **p < 0.01 by unpaired *t* test. **F** A representative NIK immunoblot from n = 3.

In our recent RNAseq analyses, we found a cohort of over 300 common genes that are upregulated by sorafenib and concurrently repressed by DDX5 [7]. KEGG pathway analysis of these 300 common genes identified the top 10 predicted cellular pathways including the Wnt signaling pathway that we have previously described [7], and the NF-kB pathway [7]. In this study, our focus was on key upregulated genes involved in the non-canonical NF-kB pathway [22], namely, *MAP3K14, NFKB2*, and *RelB* (Fig. 1A).

To validate this observation, we quantified *NIK* mRNA and protein levels in HepAD38 [30] and Huh7 cells, subjected to sorafenib treatment. Downregulation of DDX5 by siRNA targeting DDX5 (siDDX5) or sorafenib treatment resulted in increased *NIK* mRNA (Fig. 1B) and protein levels in both HepAD38 and Huh7 cells (Fig. 1C and Supplementary Fig. S1A). Conversely, DDX5 overexpression (DDX5^OE^) by Dox addition [7] fully suppressed sorafenib-induced *NIK* mRNA (Fig. 1B) and protein levels (Fig. 1C and Supplementary Fig. S1A), linking the sorafenib-mediated NIK induction to the downregulation of DDX5. Sorafenib also enhanced *NIK* mRNA expression in Huh7 xenograft tumors (Supplementary Fig. S1B and Fig. 1D). Notably, in Huh7-DDX5^KO^ cells [7], the expression of both *NIK* mRNA (Fig. 1E) and protein (Fig. 1F) was not further enhanced upon sorafenib addition, supporting the role of DDX5 in regulating NIK expression.

**Fig. 2:**
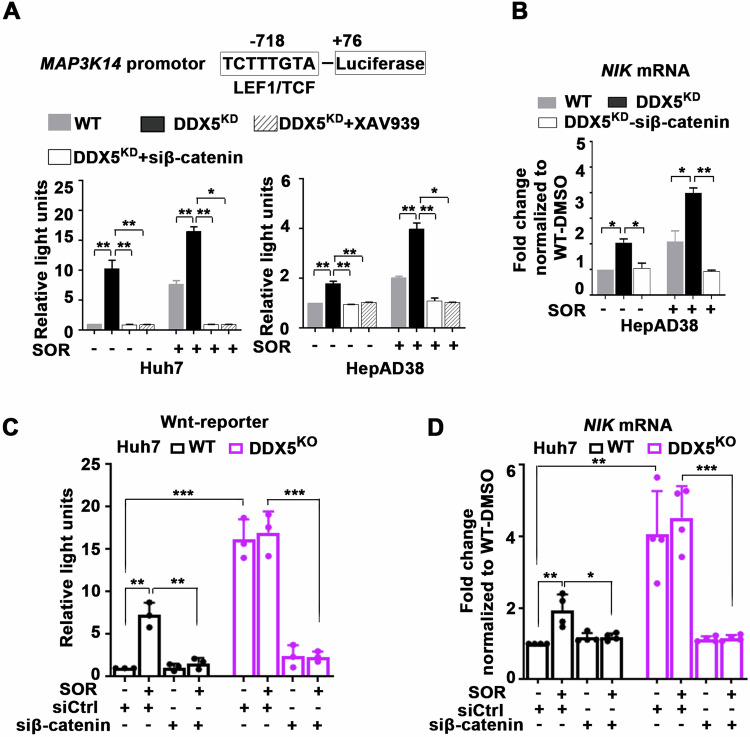
DDX5 via Wnt activation regulates non-canonical NF-κB signaling in sorafenib treated cells. **A** WT and DDX5^KD^ Huh7 and HepAD38 cells co-transfected with *MAP3K14-*firefly luciferase and Renilla-luciferase (100 ng each plasmid per 12-well plate) ±SOR (10 µM), XAV939 (20 µM) or siβ-catenin (50 pM), as indicated. Data expressed as mean ± SEM, n = 3. *p < 0.05, ** p < 0.01 by unpaired *t* test. **B** qRT-PCR of *NIK* mRNA using total RNA from WT and DDX5^KD^ HepAD38 transfected with siβ**-**catenin (50 pM) and treated ± SOR (10 µM). Data expressed as mean ± SEM, n = 3. *p < 0.05, ** p < 0.01 by unpaired *t* test. **C** Huh7 WT and DDX5^KO^ cells co-transfected with Wnt-reporter (TopFlash) and Renilla-luciferase (100 ng each plasmid per 12-well plate), and siRNAs (50 pM each) siCtrl or siβ-catenin, ±SOR (10 µM) for 24 h. Data expressed as mean ± SEM, n = 3. **p < 0.01 and ***p < 0.001 by unpaired *t* test. **D** qRT-PCR of *NIK* mRNA using total RNA from WT and DDX5^KO^ Huh7 cells, transfected with siβ-catenin (50 pM), and treated ±SOR (10 µM), as indicated. Data expressed as mean ± SEM, n = 3. *p < 0.05, ** p < 0.01 ***p < 0.001 by unpaired *t-*test.

To elucidate the mechanism by which DDX5 deficiency induces *NIK* transcription, we explored the role of the Wnt/β-catenin pathway. Analysis of the *MAP3K14* promoter revealed a putative LEF/TCF binding site at position -718, suggesting potential regulation by Wnt/β-catenin signaling, activated by DDX5 downregulation [7]. Consistent with the hypothesis of Wnt/β-catenin involvement, the *MAP3K14*-luciferase reporter was activated in DDX5^KD^ and sorafenib-treated HepAD38 and Huh7 cells (Fig. 2A). By contrast, *MAP3K14*-luciferase expression was fully repressed by pharmacologic (XAV939) or genetic (siβ-catenin) inhibition of Wnt/β-catenin signaling (Fig. 2A). Moreover, the critical role of Wnt/β-catenin signaling in *NIK* transcription was demonstrated by the reduction of *NIK* mRNA levels following siRNA-mediated knockdown of β-catenin (Fig. 2B).

Extending our investigation to Huh7-DDX5^KO^ cells, we observed increased expression driven from the *MAP3K14*-luciferase reporter (Fig. 2C) and elevated *NIK* mRNA levels (Fig. 2D), regardless of sorafenib addition. Conversely, inhibition of Wnt signaling by siβ-catenin suppressed both *MAP3K14*-luciferase reporter (Fig. 2C) and *NIK* mRNA levels (Fig. 2D). Together, these results demonstrate that activation of Wnt/β-catenin signaling by DDX5 deficiency induced *NIK* transcription.

### DDX5 downregulation promotes activation of non-canonical NF-κB signaling

**Fig. 3:**
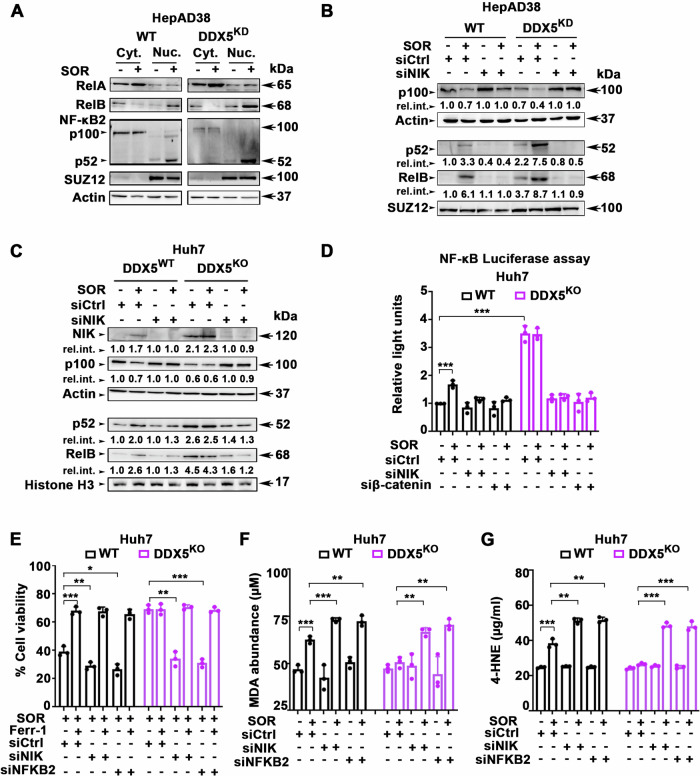
Activation of Non-canonical NF-κB mediates ferroptosis escape by sorafenib. Immunoblots with indicated antibodies, using cytoplasmic and nuclear lysates from WT and DDX5^KD^ HepAD38 cells treated ±7.5 µM SOR for 2 days (**A**) and transfected with 50 pM each siCtrl (-) or siNIK, as indicated, in (**B**). Actin used as loading control for cytoplasmic lysates and SUZ12 for nuclear extracts. **C** WT and DDX5^KO^ Huh7 cells treated ±10 µM SOR for 2 days and transfected with 50 pM each siCtrl (-) or siNIK, as indicated. A representative immunoblot is shown from n = 3. Actin used as loading control for cytoplasmic lysates and Histone 3 for nuclear extracts. **D** NF-κB-response element (RE) luciferase vector (pNL3.2.NF-κB-RE vector, Promega) (1.0 µg per 12-well plate) co-transfected in Huh7 WT or DDX5^KO^ cells with 50 pM each of siCtrl, siNIK or siβ-catenin, ±SOR (10 µM) for 48 h. Data expressed as mean ± SEM from n = 3. ***p < 0.001 by unpaired *t-*test. **E** Cell viability of Huh7 WT or DDX5^KO^ cells transfected with indicated siRNAs (50 pM) treated with SOR (10 µM) ±10 Ferr-1 (10 µM) for 24 h. Data expressed as mean ± SEM from n = 3. *p < 0.05, **p < 0.01, ***p < 0.001 by unpaired *t-*test. **F** MDA and (**G**) 4-HNE abundance quantified using lysates from Huh7 WT and DDX5^KO^ cells, transfected with indicated siRNAs (50 pM), treated with SOR (10 µM) ±10 Ferr-1 (10 µM) for 24 h. Data expressed as SD, n = 3. *p < 0.05, **p < 0.01, ***p < 0.001 by unpaired *t-*test.

A hallmark of non-canonical NF-κB signaling activation is the de novo synthesis and accumulation of NIK, which mediates the processing of NFKB2/p100 to p52, followed by nuclear accumulation with RelB [22]. Sorafenib promoted the nuclear accumulation of p52 and RelB, but not RelA, a key protein involved in canonical NF-κB signaling (Fig. 3A). Silencing NIK expression by siRNA (siNIK) inhibited sorafenib-induced processing of NFKB2/p100 and the nuclear accumulation of p52/RelB (Fig. 3B). Significantly, Huh7-DDX5^KO^ cells exhibited constitutive expression of NIK and increased accumulation of p52/RelB in the nucleus, independent of sorafenib addition (Fig. 3C). Knockdown of NIK expression by siNIK, restored cytoplasmic p100 protein levels and reduced the nuclear accumulation of p52 and RelB (Fig. 3C).

To further delineate how DDX5 downregulation induced activation of non-canonical NF-κB signaling, we performed NF-κB-luciferase assays in both WT and DDX5^KO^ Huh7 cells (Fig. 3D). Sorafenib increased NF-κB luciferase activity in WT Huh7 cells, while Huh7 DDX5^KO^ cells exhibited constitutive activation of the NF-κB-luciferase reporter. Transfection of siNIK or siβ-catenin in Huh7 DDX5^KO^ cells, treated with or without sorafenib, completely inhibited NF-κB-luciferase expression, indicating activation of the non-canonical NF-κB pathway is NIK-dependent, induced by Wnt signaling (Fig. 3D). Similarly, in WT HepAD38 and Huh7 cells, treatment with the NIK inhibitor B022 or DDX5^OE^ fully repressed NF-κB-luciferase activation induced by siDDX5 or in combination with sorafenib (Supplementary Fig. S2A). These findings collectively support that DDX5 is an upstream negative regulator of non-canonical NF-κB signaling.

**Fig. 4:**
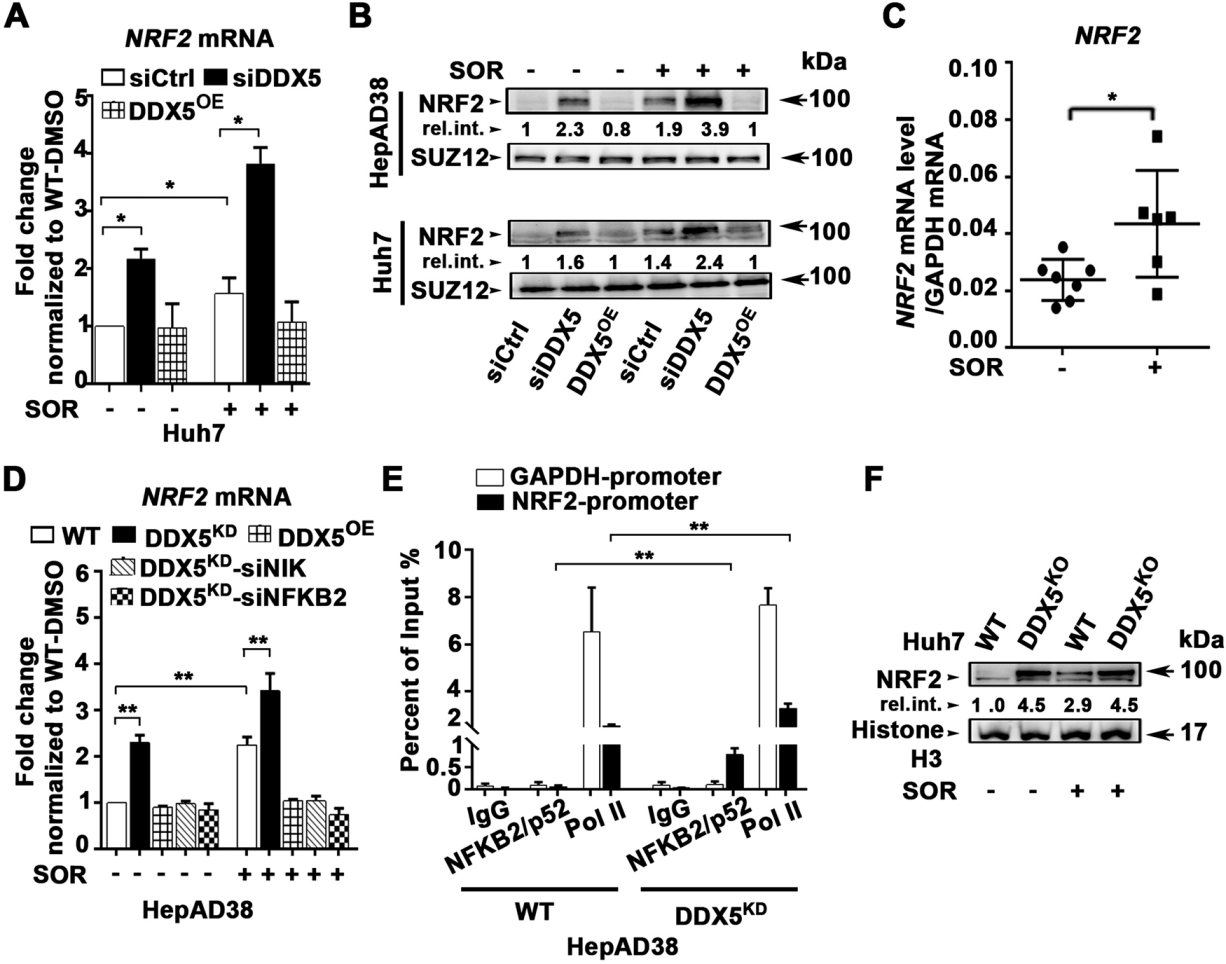
Activation of non-canonical NF-κB by DDX5 downregulation mediates *NRF2* transcription. **A** qRT-PCR of *NRF2* mRNA using total RNA isolated from Huh7 cells transfected with siCtrl or siDDX5 (50 pM) and Huh7 DDX5^OE^ cells, treated ±SOR (10 µM) for 24 h. Data expressed as mean ± SEM from n = 3. *p < 0.05 by unpaired *t-*test. **B** Immunoblots of NRF2 using nuclear lysates from Huh7 and HepAD38 cell lines transfected with siCtrl or siDDX5 ±10 µM SOR for 24 h. For DDX5^OE^, Dox-inducible-DDX5 cell lines grown with Dox (1.0 µg/ml) for 48 h and SOR for the last 24 h. A representative immunoblot is shown from n = 3. **C** RT-PCR quantification of *NRF2* mRNA using total RNA isolated from Huh7 xenograft tumors, treated ±SOR. Data expressed as mean ± SEM from eight tumors described in Li et al. [7]. *p < 0.05 by unpaired *t-*test. **D** qRT-PCR of *NRF2* mRNA using total RNA isolated from HepAD38 WT, DDX5^KD^, and DDX5^OE^ cells, transfected with indicated siRNAs (50 pM each) and treated +/- SOR (10 µM) for 24 h. Data expressed as mean ± SEM from n = 3. **p < 0.01 by unpaired *t-*test. **E** ChIP assays of *NRF2* and *GAPDH* promoters using IgG, NFKB2/p52 or RNA polymerase II (Pol II) antibodies, in WT and DDX5^KD^ HepAD38 cells. Data expressed as mean ± SEM from two independent experiments. **p < 0.01 by unpaired *t-*test. **F** Immunoblots of NRF2 using WT and DDX5^KO^ Huh7 cells treated ±SOR (10 μM) for 48 h. A representative immunoblot from n = 3.

To assess the involvement of the non-canonical NF-κB pathway in ferroptosis escape induced by DDX5 loss in sorafenib-treated HCC cells, we examined the effect of siNIK and siNFKB2 using cell viability assays. In both WT and DDX5^KO^ Huh7 cells treated with sorafenib, siNIK, and siNFKB2 significantly reduced cell viability. Importantly, this reduction in cell viability was effectively restored by addition of ferrostatin (Ferr-1), an inhibitor of feroptosis (Fig. 3E). To further establish the effect on ferroptosis, we measured the levels of lipid peroxidation by-products malondialdehyde (MDA) and 4-hydroxynonenal (4-HNE), known hallmarks of ferroptosis [34, 35]. In sorafenib-treated WT and DDX5^KO^ Huh7 cells, siNIK or siNFKB2 led to substantial increase in MDA (Fig. 3F) and 4-HNE levels (Fig. 3G). This observed elevation in lipid peroxidation by-products indicates a pro-ferroptotic effect by inhibiting the non-canonical NF-κB pathway. Similar results were observed in WT and DDX5^KD^ HepAD38 cells (Supplementary Fig. S2B–D). Collectively, these findings identify a previously unknown function of the non-canonical NF-κB pathway in ferroptosis escape. Importantly, they demonstrate the crucial role of DDX5 in modulating the ferroptotic response to sorafenib through NIK-mediated activation of non-canonical NF-κB (Fig. 3).

### Non-canonical NF-κB mediates ferroptosis escape by inducing *NRF2* transcription

To further investigate the role of the non-canonical NF-κB in ferroptosis escape, we focused on NRF2, a key player in cytoprotective gene transcription during cellular stress [17]. Previous studies reported NRF2 “activation” in response to sorafenib, through an unidentified mechanism [6]. Significantly, NRF2 emerged as one of the genes induced by sorafenib and repressed by DDX5 in our RNAseq analyses (Fig. 1B).

Indeed, both DDX5 downregulation and sorafenib treatment led to increased *NRF2* mRNA levels (Fig. 4A) and protein levels (Fig. 4B), while DDX5^OE^ fully repressed this induction. Consistently, sorafenib increased transcription of *NRF2* in Huh7 xenograft tumors (Supplementary Fig. S1B and Fig. 4C). Importantly, siRNA-mediated knockdown of *NIK* or *NFKB2* fully repressed *NRF2* expression, suggesting non-canonical NF-κB is required for *NRF2* transcription (Fig. 4D).

To validate this hypothesis, we performed ChIP assays using anti-NFKB2/p52 in both WT and DDX5^KD^ HepAD38 cells. The *NRF2* promoter exhibited increased occupancy by the pNFKB2/p52 subunit and RNA polymerase II in DDX5^KD^ cells, compared to the control GAPDH promoter (Fig. 4E). This evidence demonstrates that non-canonical NF-κB (p52/RelB) mediates *NRF2* transcription. Importantly, in Huh7-DDX5^KO^ cells, NRF2 expression similar to the expression of NIK (Fig. 1E, G), is independent of sorafenib addition (Fig. 4F).

### DDX5 regulates NRF2 protein stability

**Fig. 5:**
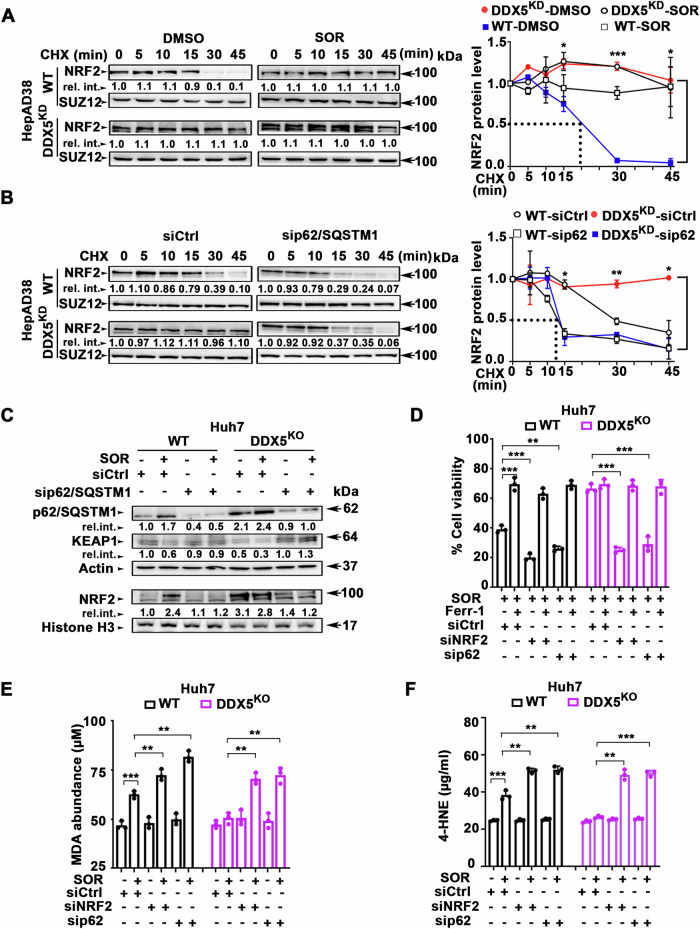
DDX5 downregulation enhances NRF2 protein stability and ferroptosis escape by sorafenib. **A** Immunoblots of NRF2 using WT and DDX5^KD^ HepAD38 cells treated with DMSO or SOR (7.5 μM) for 48 h, followed by treatment with MG132 (10 μM for 4 h), extensive washing, and addition of cyclohexamide (CHX, 200 µM), as indicated. Nuclear extracts were isolated following the time course of CHX treatment and immunoblotted with indicated antibodies. A representative immunoblot from three independent experiments. *Right panel* Quantification of NRF2 t1/2 by ImageJ software. Error bars represent SD from n = 3. *p < 0.05, ***p < 0.001 by unpaired *t-*test. **B** Immunoblots of NRF2 in sip62/SQSTM-transfected cells. Lysates prepared from WT and DDX5^KD^ HepAD38 cells transfected with 50 pM siRNAs (siCtrl or sip62/SQSTM1) for 48 h, followed by addition of MG132 (10 μM for 4 h), extensive washing, and addition of CHX (200 µM) for the indicated intervals. Nuclear lysates subjected to immunoblot analysis. A representative immunoblot is shown from n = 3. *Right panel* Quantification of NRF2 t1/2 by ImageJ software. Error bars represent SD from n = 3. *p < 0.05, **p < 0.01 by unpaired *t-*test. **C** Immunoblots with indicated antibodies using lysates from WT and DDX5^KO^ Huh7 cells treated ±SOR for 24 h. A representative experiment is shown from n = 3. **D** Cell viability of WT and DDX5^KO^ Huh7 cells transfected with indicated siRNAs (50 pM), treated with SOR (10 µM) ± Ferr-1 (10 µM) as indicated, for 24 h. Data expressed as mean ± SEM from n = 3. **p < 0.01, p*** < 0.001 by unpaired *t-*test. **E** MDA and (**F**) 4-HNE abundance quantified using lysates from Huh7 WT and DDX5^KO^ cells, transfected with indicated siRNAs (50 pM), ±SOR (10 µM) for 24 h. Data expressed as SD, n = 3. **p < 0.01, ***p < 0.001 by unpaired *t-*test.

Sorafenib treatment as well as DDX5^KD^ independently extended NRF2 half-life to over 45 min (Fig. 5A), suggesting a correlation between DDX5 and NRF2 protein stability. To elucidate this mechanism, we investigated the involvement of p62/SQSTM1, a critical factor in NRF2 stabilization, acting through KEAP1 inactivation [17]. Silencing of p62/SQSTM1 by siRNA (sip62/SQSTM1) in DDX5^KD^ cells significantly reduced NRF2 half-life to approximately 15 min, abolishing the extended NRF2 half-life observed in the absence of DDX5 (Fig. 5B).

In comparison to WT Huh7 cells, DDX5^KO^ cells displayed increased levels of p62/SQSTM1, reduced KEAP1, and elevated NRF2. Notably, Huh7-DDX5^KO^ exhibited sorafenib–independent deregulation of these proteins, when compared to WT Huh7 cells (Fig. 5C and Supplementary Fig. S3). The enhanced NRF2 protein levels in DDX5^KO^ cells correlated with increased p62/SQSTM1 protein levels (Fig. 5C and Supplementary Fig. S3), in agreement with prior findings, indicating that DDX5 reduces p62/SQSTM1 stability [21]. Indeed, siRNA-mediated knockdown of p62/SQSTM1 increased KEAP1 levels, while reducing NRF2 protein levels (Fig. 5C). In summary, the loss of DDX5 augments p62/SQSTM1 protein levels, leading to KEAP1 inactivation and consequent enhancement of NRF2 stability.

Since DDX5 downregulation induces ferroptosis escape of sorafenib-treated cells [7] enhancing both *NRF2* transcription and protein stability (Fig. 5A–C), we next examined the effect of NRF2 knockdown on ferroptosis escape. NRF2 knockdown (siNRF2) or p62/SQSTM1 knockdown (sip62) effectively inhibited ferroptosis escape of sorafenib-treated WT and DDX5^KO^ Huh7 cells, determined by cell viability assays (Fig. 5D). These knockdowns also increased the formation of lipid peroxidation by-products MDA (Fig. 5E) and 4-HNE (Fig. 5F), hallmarks of ferroptosis. Similar results were observed in WT and DDX5^KD^ HepAD38 cells (Supplementary Fig. S4A–C). Together, these results demonstrate the dual role of DDX5 downregulation by sorafenib in orchestrating NRF2 activation and ferroptosis escape; namely, enhancement of both *NRF2* transcription and NRF2 protein stability.

### Knockdown of NRF2 or overexpression of DDX5 in xenograft tumors enhanced anti-tumor efficacy of sorafenib

To investigate the significance of NIK/non-canonical NF-κB pathway as an effector of ferroptosis escape and sorafenib response in vivo, we utilized the Huh7 xenograft mouse model. Due to the absence of stable NIK inhibitors for in vivo studies and the lack of direct NRF2 targeting inhibitors [36], we employed RNA interference. Since NRF2 is the downstream target of the non-canonical NF-kB pathway (Fig. 4), we developed an in vivo siRNA-mediated inhibition of *NRF2* mRNA using the recently developed Nanosac, a polyphenol nanocapsule [32], as siRNA carrier. We selected the Nanosac-encapsulated siRNA delivery, as proof-of-concept method, because of the effective intracellular release of siRNA without endosomal sequestration [32].

**Fig. 6:**
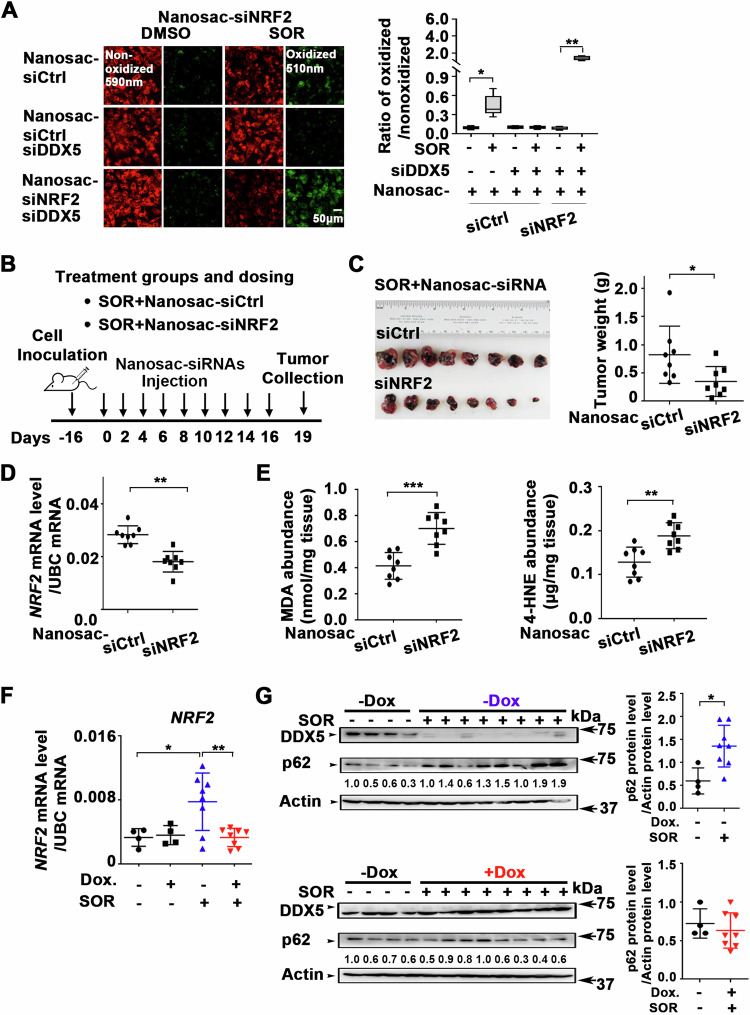
Nanosac-encapsulated siRNA targeting *NRF2* enhances antitumor efficacy of sorafenib. **A** Fluorescence microscopy of C11-BODIPY using Huh7 cells transfected with siDDX5 and incubated with Nanosac-encapsulated siCtrl or siNRF2 for 24 h, followed by addition of SOR (10 μM) for 24 h. Quantification by ImageJ software of the ratio of oxidized (510 nm)/non-oxidized (590 nm) C11-BODIPY. Data shown as mean ± SEM from 500 cells. *p < 0.05, **p < 0.01 by unpaired *t-*test. **B** Diagram illustrates treatment groups and timetable of intratumoral injection of Nanosac-encapsulated siRNAs. **C** Images of Huh7 xenograft tumors excised on day 19, following three intra-tumoral injections/week of indicated Nanosac-encapsulated siRNAs (3.0 µg siRNA/injection), and daily administration of SOR (80 mg/kg). Tumor weight of indicated treatment groups from eight tumors. *p < 0.05 by unpaired *t-*test. **D** RT-PCR quantification of *NRF2* mRNA using total RNA isolated from Huh7 tumors treated with indicated Nanosac-encapsulated siRNAs ±SOR. Data expressed as mean ± SEM from eight tumors. *p < 0.05 by unpaired *t-*test. **E** Quantification of MDA and 4-HNE abundance using Huh7 xenograft tumors treated as indicated. Data expressed as mean ± SEM from eight tumors. **p < 0.01, ***p < 0.001 by unpaired *t-*test**. F** RT-PCR quantification of *NRF2* mRNA using total RNA isolated from Dox-inducible Huh7-DDX5 tumors, -/+ Dox and SOR. Data expressed as mean ± SEM from indicated number of tumors in each group. *p < 0.05, **p < 0.01 by unpaired *t-*test. **G** Immunoblots of DDX5 and p62/SQSTM1 using lysates from Dox-inducible Huh7-DDX5 tumors, -**/**+ Dox and SOR administration (80 mg/kg, 5 days per week), as described in Li et al., [7]. Quantification by imageJ software of p62/SQSTM1. Data expressed as mean ± SEM from eight tumors. *p < 0.05 by unpaired *t-*test.

Nanosac-encapsulated siRNA targeting *NRF2* induced ferroptosis in siDDX5 transfected cells treated with sorafenib, quantified by C11-BODIPY (Fig. 6A). C11-BODIPY is a lipid-soluble fluorescent indicator of lipid peroxidation and ferroptosis [37]. Next, we generated Huh7 xenografts in immunocompromised NRG mice and after tumor formation, animals were co-treated with sorafenib and Nanosac-encapsulated siRNAs (siCtrl or siNRF2), as illustrated in Fig. 6B. Intra-tumoral injection of Nanosac-siNRF2 in combination with sorafenib significantly reduced tumor weight (Fig. 6C) and *NRF2* mRNA levels, compared to Nanosac-siCtrl (Fig. 6D). The elevated levels of MDA and 4-HNE quantified in sorafenib and siNRF2 treated tumors (Fig. 6E), both recognized in vivo markers of lipid peroxidation and ferroptosis [35], indicate siRNA interfering with NRF2 expression enhances the anti-tumor efficacy of sorafenib in vivo.

Next, we analyzed Huh7 xenograft tumors generated using the Dox-inducible Huh7-DDX5 cell line [7]. Animals bearing tumors were treated with Dox-containing H_2_O 48 h before sorafenib administration, and this treatment continued for 10 days. In the group of Huh7 xenograft tumors from Dox-fed animals treated with sorafenib, we observed increased DDX5 protein levels accompanied by a reduction in tumor size [7]. Importantly, sorafenib induced *NRF2* mRNA levels in xenograft tumors grown without Dox, whereas ectopic, Dox-induced DDX5 expression suppressed this *NRF2* mRNA induction (Fig. 6F). Similarly, Dox-induced DDX5 expression increased MDA and 4-HNE levels in tumors treated with sorafenib [7].

To validate the role of DDX5 in suppressing NRF2 expression in xenograft tumors, we assessed the protein level of p62/SQSTM1, a known upstream positive regulator of NRF2 protein stability, using immunoblots. In xenograft tumors grown in the absence of Dox (-Dox), sorafenib reduced endogenous DDX5 levels, while increasing p62/SQSTM1 protein levels. In contrast, the combination of sorafenib with Dox-induced DDX5 expression did not increase p62/SQSTM1 levels (Fig. 6G). Based on our in vitro mechanistic analyses (Fig. 5), we interpret these results to indicate that elevated p62/SQSTM1 levels in the absence of DDX5 (Fig. 6G), lead to increased NRF2 stability. Conversely, ectopic induction of DDX5 with Dox in the presence of sorafenib suppressed this increase in p62/SQSTM1 protein levels [21], leading to decreased NRF2 stability. These results elucidate the mechanism by which DDX5 orchestrates NRF2 activation in vivo and underscore the therapeutic potential of siRNA-mediated therapies targeting *NRF2* in combination with sorafenib for the effective treatment of HCC.

### Decreased DDX5 protein levels in human HCCs are linked with activation of non-canonical NF-κB

**Fig. 7:**
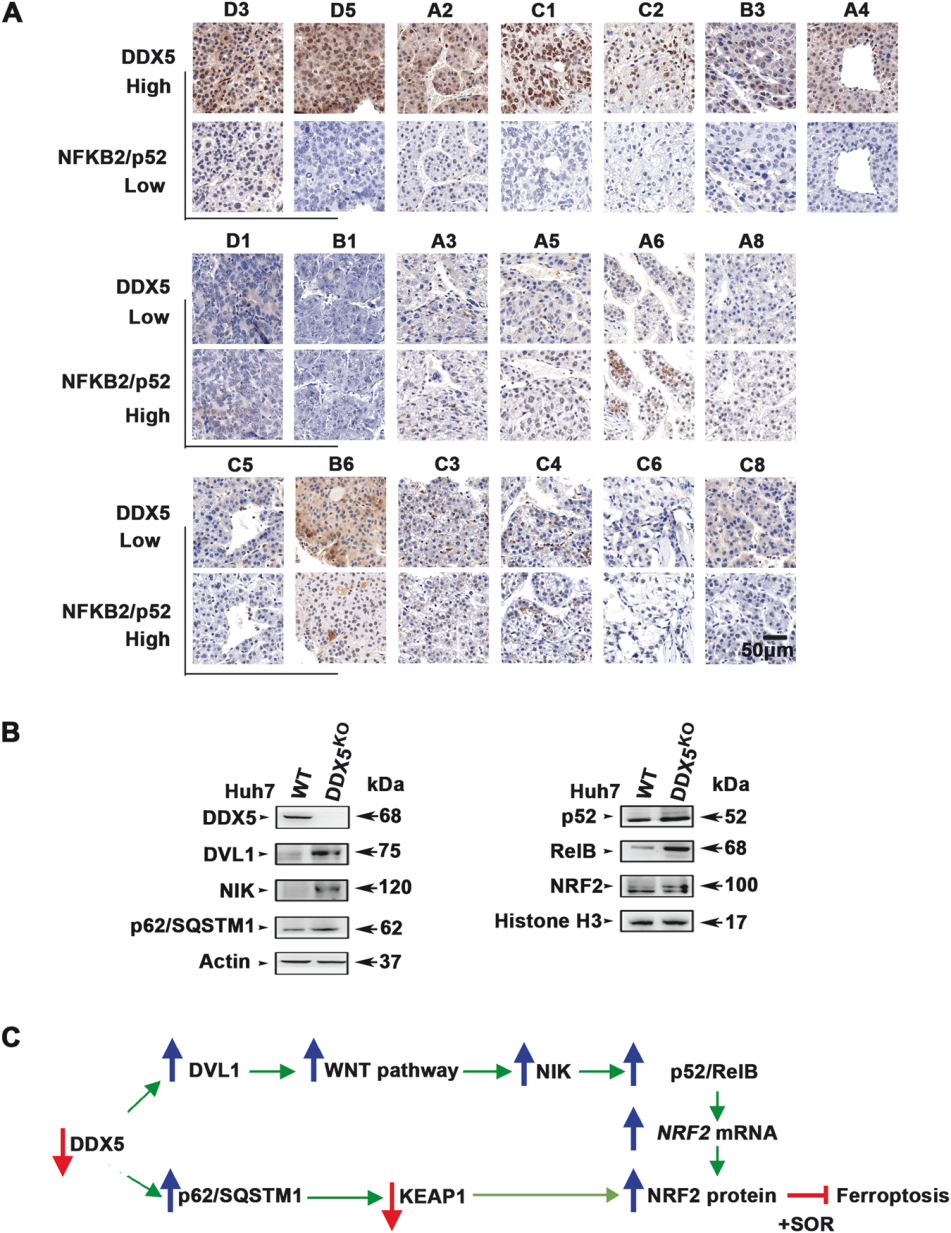
Reduced DDX5 protein levels in human HCCs associated with increased nuclear NFKB2/p52. **A** Immunohistochemistry (IHC) of human HCCs from tissue microarray (TMA) referenced in [7], using DDX5 and NFKB2/p52 antibodies. The numbers above each image indicate the tumor position in TMA (Supplementary Fig. S4). Representative images at 20X magnification. **B** Immunoblot of lysates from WT and DDX5^KO^ cells using the indicated antibodies. Histone 3 is loading control for nuclear lysates. **C** Diagram illustrates the interconnected pathways activated by DDX5 downregulation. Specifically, decreased levels of DDX5 lead to activation of Wnt/β-catenin signaling by inducing DVL1, resulting in transcriptional upregulation of NIK and subsequent activation of non-canonical NF-κB (p52/RelB). This activation leads to transcriptional induction of *NRF2*. Additionally, DX5 deficiency stabilizes p62/SQSTM1, leading to proteasomal degradation of KEAP1, thus enhancing NRF2 stabilization.

To further establish the clinical relevance of our mechanistic results, we investigated the association between DDX5 protein levels and non-canonical NF-κB activation, using a tissue microarray (TMA) comprised of grades I-III human HCCs. In our earlier studies, employing DDX5 immunohistochemistry of the same TMA, we observed positive nuclear staining of DDX5 in normal liver and grade I tumors, while HCC grades II-III exhibited a reduced number of DDX5-positive staining cells [7]. Here, we observe, an inverse relationship between DDX5 expression and positive immunostaining for the nuclear NFKB2/p52 subunit of non-canonical NF-κB (Fig. 7A and Supplementary Fig. S5). Significantly, these HCC samples were obtained from patients who had not undergone sorafenib treatment, reinforcing the constitutive expression of NIK/p52/RelB and NRF2 observed in the Huh7-DDX5^KO^ cells (Fig. 7B). Collectively, these findings support a model in which deficiency of DDX5 through Wnt/β-catenin activation [7] induces non-canonical NF-κB (p52/RelB), leading to ‘activation” of NRF2 (transcription and stabilization), ultimately driving ferroptosis escape of sorafenib-treated cells, as illustrated by the diagram (Fig. 7C).

### Transcriptomic analyses of liver tumors with reduced DDX5

Recent studies have identified a significant decrease in DDX5 expression in preclinical mouse models transitioning from NASH to HCC [26]. To explore whether this downregulation of DDX5 during NASH progression to HCC activates the same pathways associated with enhanced expression of DDX5-regulated genes (Fig. 7B), we analyzed the transcriptomic data from the Stelic Animal Model (STAM), which recapitulates NASH-induced HCC [38]. We opted for the STAM dataset due to its molecular similarity to human HCCs, as demonstrated in prior integrative transcriptomic and genomic investigations [28]. Our bioinformatics analyses of the RNAseq data from the STAM model [28, 29] revealed a reduction in *Ddx5* expression, along with the upregulation of Wnt signaling genes (*Dvl1, Dvl2, Dvl3*, and *Axin1*), consistent with our recent findings [7]. Additionally, we observed an elevation in non-canonical NF-κB pathway genes (*Map3k14, Relb*, and *NfkB2*), alongside *Nrf2* and its downstream target genes *Sqstm1*, *Nqo1* [17, 39] and *Spp1* [40]. Significantly, *SPP1* encoding osteopontin is closely linked to tumor cell evolution/heterogeneity and poor patient prognosis [41].

**Fig. 8:**
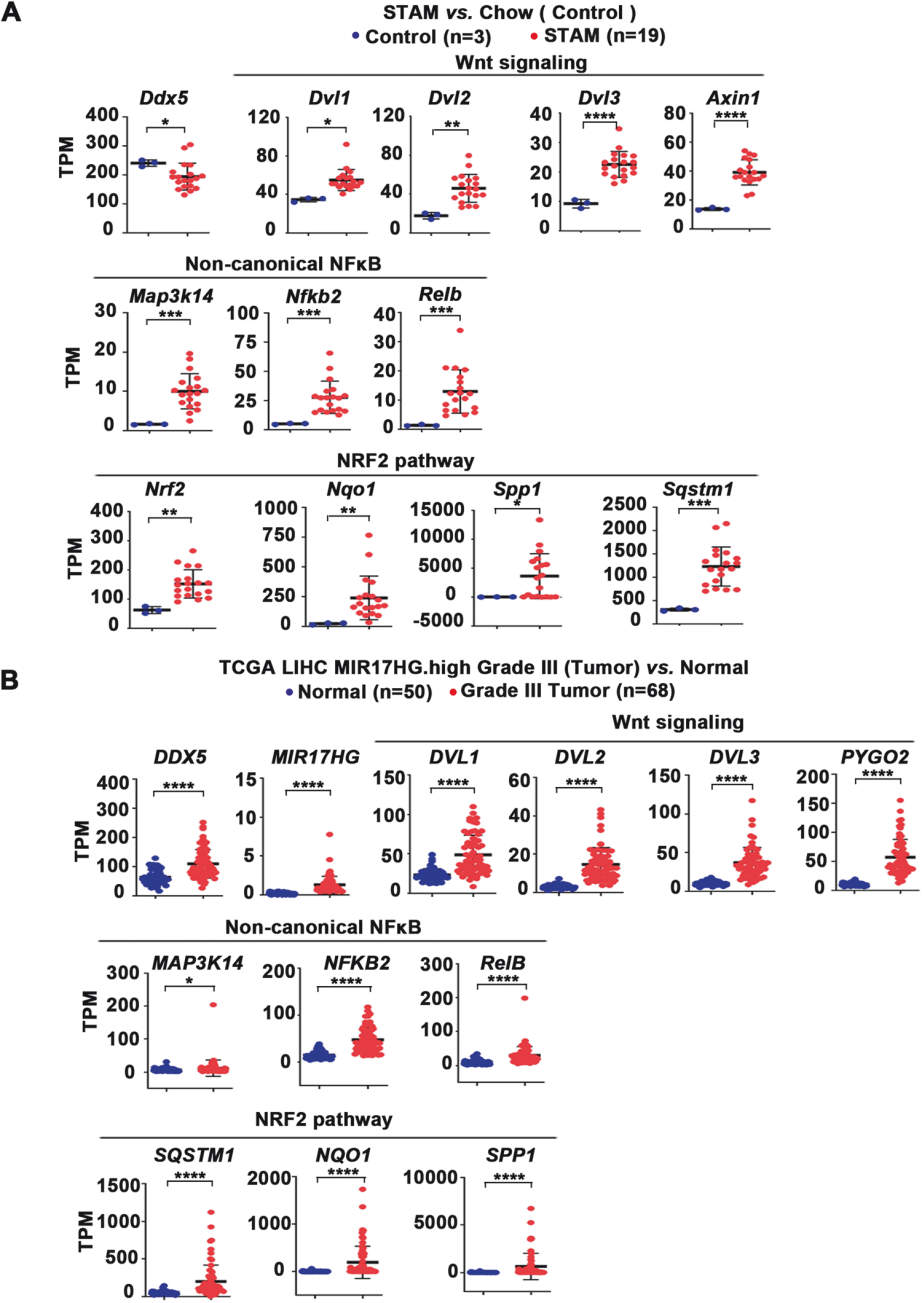
Elevated expression of Wnt/β-catenin signaling, non-canonical NF-κB, and NRF2 pathways/genes. **A** Analyses of transcriptomic data from STAM vs. Chow described in [28, 29]. Plots showing the expression (TPM) of genes associated with activation of Wnt/β-catenin signaling, non-canonical NF-κB, and NRF2 in livers of STAM vs. Chow-fed mice. **B** Analyses of transcriptomic data from human HCCs expressing *MIR17HG*.high, grade III (n = 68) *vs*. normal liver (n = 50) from TCGA.LIHC. *MIR17HG*.high samples are defined by stratifying TCGA.LIHC patients (tumor data only) into 4 quantiles by expression (TPM) for *MIR17HG*. Patients stratified into top two quantiles (n = 185) were denoted as “*MIR17HG*.high” group (Table S5). Plots showing the expression (TPM) of genes associated with activation of Wnt/β-catenin signaling, non-canonical NF-κB, and NRF2. Error bars indicate mean ± SEM. *p < 0.05; **p < 0.01; ***p < 0.001; ****; p < 0.0001 by unpaired *t-*test.

Next, we performed transcriptomic analyses comparing normal liver samples to HCCs from TCGA, focusing on HCCs likely exhibiting decreased DDX5 expression. Our criteria for selecting these HCC samples were based on several factors: (i) the demonstration of reduced DDX5 protein levels in grades II-III HCCs [7]; (ii) the association of *MIR17HG* RNA, which encodes the proto-oncogenic miR17 ~ 92 cluster, with poor prognosis HCC [42, 43]; and (iii) the regulatory role of miR17 ~ 92 and its paralog miR106b ~ 25 in suppressing the expression of several tumor suppressors, including DDX5 [11]. Based on these considerations, we compared the transcriptome of *MIR17HG*-high grade-III HCCs from TCGA to normal liver (Supplementary Table S5). This analysis revealed increased expression of genes associated with Wnt signaling activation, concurrent with the activation of non-canonical NF-κB and NRF2 pathways (Fig. 8B). Based on our recent studies [7] and the results shown herein (Figs. 1, 2, 4, 7 and 8), the activation status of these three interconnected pathways, i.e., Wnt/non-canonical NF-κB/NRF2, supports the downregulation of DDX5 in advanced HCC, emphasizing the therapeutic potential of targeting these interconnected pathways.

## Discussion

Our recent study demonstrated the critical role of the RNA helicase DDX5 in determining sorafenib efficacy in the treatment of HCC [7]. Sorafenib, as well as other multi-tyrosine kinase inhibitors, were shown to induce the downregulation of DDX5 in HCC cell lines and preclinical HCC models. Clinical data also support these mechanistic results, revealing a correlation between reduced DDX5 protein levels, advanced tumor grade, and poor patient survival post-sorafenib treatment [7].

DDX5 is an upstream negative regulator of Wnt signaling genes and Wnt activation [7]. Wnt signaling has a fundamental role in all aspects of liver development, regeneration [44], and HCC pathogenesis [15] including cancer drug resistance [45–47]. The downregulation of DDX5 by sorafenib, as observed in our studies, activates Wnt/β-catenin signaling leading to ferroptosis escape [7], a mechanism implicated in acquired cancer drug resistance [12, 13]. In this study, we investigated the impact of Wnt/β-catenin activation due to DDX5 deficiency in ferroptosis escape of sorafenib-treated HCC cells and HCC progression.

### DDX5 deficiency promotes activation of non-canonical NF-κB and NRF2 pathways, leading to ferroptosis escape of sorafenib-treated HCC cells

Our RNA-seq analyses [7] identified genes induced by sorafenib and concurrently repressed by DDX5, such as *DVL1* [7], crucial for Wnt activation, and NIK (Fig. 1B), essential for non-canonical NF-κB pathway activation [20]. Our investigations (Fig. 2) demonstrated that the *MAP3K14* promoter contains a functional LEF/TCF site, establishing a direct connection between Wnt/β-catenin activation and *NIK* transcription. Indeed, *NIK* expression by DDX5 downregulation was abrogated by pharmacologic (XAV939) or genetic (siβ-catenin) inhibition of Wnt signaling (Fig. 2). Moreover, both NIK expression and Wnt/β-catenin activation are required for NF-κB-reporter expression (Fig. 3). Silencing of NIK or NFKB2/p100 suppressed ferroptosis escape of sorafenib-treated cells, reinforcing the mechanistic link between DDX5 downregulation, non-canonical NF-κB activation, and ferroptosis escape (Fig. 3), as illustrated in Fig. 7C.

The connection between non-canonical NF-κB (p52/RelB) activation and ferroptosis evasion of sorafenib-treated/DDX5 downregulated cells involves induction of *NRF2* transcription (Fig. 4). Furthermore, the reduction in DDX5 has an additional effect on NRF2, extending its half-life through stabilization of p62/SQSTM1 [21], thereby inactivating KEAP1 (Fig. 5). KEAP1, identified as a sorafenib-sensitivity gene in HCC [18], mediates NRF2 proteasomal degradation [16]. Thus, DDX5 deficiency exerts a dual effect on sorafenib sensitivity by enhancing both *NRF2* transcription and protein stabilization, thereby influencing ferroptosis susceptibility (Figs. 5 and 8).

Importantly, siRNA-mediated knockdown of *NRF2* emerges as an effective strategy to improve the antitumor effectiveness of sorafenib/mTKIs (Fig. 6). The overexpression of DDX5 in Huh7 xenografts not only suppressed tumor growth following sorafenib treatment [7] but also repressed the protein level of p62/SQSTM1, a positive upstream effector of NRF2 stability (Fig. 6). This molecular understanding of the key players in sorafenib sensitivity identifies potential therapeutic targets for enhancing antitumor response of mTKIs. The continuous progress in RNA therapeutics, and the feasibility of effecient lipid nanoparticle delivery to hepatocytes [48–50], offer the possibility of targeting *NRF2* or *β-catenin* mRNA or both, as a viable therapeutic strategy for HCC, in combination with mTKIs.

### RNA targeted therapies for HCC, beyond sorafenib treatment

Our studies with the Huh7-DDX5^KO^ cell line revealed constitutive expression of NIK, persistent non-canonical NF-κB activation (Fig. 3), and prolonged expression of NRF2, even without sorafenib treatment (Fig. 7). Significantly, previous studies have linked elevated NRF2 and p62/SQSTM1 levels with HCC pathogenesis and progression [19, 20]. Recent studies have also shown increased nuclear localization of RelB in poor prognosis HCC [27]. Notably, we also observe increased nuclear NFKB2/p52 immunostaining in HCCs with low DDX5 expression (Fig. 7A), characteristic of advanced grade HCC [7]. In addition, DDX5 downregulation has been associated with the progression from NASH to HCC, while ectopic DDX5 overexpression impedes this progression [26]. Importantly, our analyses of the transcriptomic data from the NASH to HCC STAM model [28, 29], identified increased expression of the DDX5-dependent and interlinked genes and pathways, including *Dvl1, 2, 3* (associated with Wnt/β-catenin activation), *Map3k14, Nfkb2, Relb* (related to non-canonical NF-κB), and *Nrf2* along with NRF2-induced genes *Nqo1*, *p62/Sqstm1* and *Spp1* (Fig. 8A). Similarly, upregulation of these DDX5-dependent and interconnected pathways has also been observed in advanced grade-III human HCCs, those expressing high levels of *MIR17HG* RNA (Fig. 8B). While mRNA levels of *DDX5* remained elevated (Fig. 8B), our recent studies demonstrated reduced DDX5 immunostaining in advanced grade human HCCs [7]. Significantly, there is a marked upregulation in *MIR17HG* RNA levels from grade I to III HCCs (Supplementary Fig. S6), encoded by extrachromosomal circular DNA and correlated with poor patient prognosis [42, 43]. *MIR17HG* RNA encodes the proto-oncogenic miR17 ~ 92 cluster, which, along with its paralog miR106b-25, both elevated in human HCCs (Supplementary Fig. S6B), downregulate the expression of various tumor suppressors, including DDX5 [11]. Collectively, these findings highlight the potential significance of these DDX5-dependent and interconnected pathways that are upregulated in advanced HCC.

Given that cancer is a network-based disease, driven by various interconnected pathways, effective disruption of these pathways is crucial to limit cancer cell survival. Remarkably, the deficiency of DDX5 in hepatocytes activates the interconnected Wnt/β-catenin-NIK/p52/RelB- and NRF2 pathways, all relevant to cancer [19, 27, 41, 44]. Consequently, we propose that RNA-based therapies targeting these pathways could offer therapeutic benefits beyond the sorafenib/mTKI treatments. Specifically, siRNA-mediated interventions [48–50] designed to inhibit Wnt/β-catenin signaling, NIK/non-canonical NF-κB, and/or NRF2 expression, individually or in combination, could potentially prevent the transition from NASH to HCC, and improve the efficacy of existing HCC treatments.

### Supplementary information

Supplementary Material

Western Blots

## Data Availability

All sequencing data are available from the NCBI Gene Expression Omnibus (GEO) database (accession number GSE199092). Link: https://www.ncbi.nlm.nih.gov/geo/query/acc.cgi?acc=GSE199092.
