# Supplementary material for: DDX5 deficiency drives non-canonical NF-κB activation and NRF2 expression, influencing sorafenib response and hepatocellular carcinoma progression
Source: Cell Death Dis. 2024 Aug 9;15(8):583. doi: 10.1038/s41419-024-06977-z (PMC11315975; doi:10.1038/s41419-024-06977-z)
Supplement: Supplementary file 1 — Supplementary Material [file 41419_2024_6977_MOESM1_ESM.docx]

**Supplementary Information**

**DDX5 deficiency drives non-canonical NF-κB activation and NRF2 expression, influencing sorafenib response and hepatocellular carcinoma progression.**

Zhili Li^1, 2^, Woojun Kim^2, 3^, Sagar Utturkar^2^, Bingyu Yan^2, 4^, Nadia Atallah Lanman^2, 5^,

Bennett D Elzey^2, 5^, Majid Kazemian^2, 4, 6^, Yoon Yeo^2, 3^ and Ourania Andrisani^1,2*^

^1^Department of Basic Medical Sciences, Purdue University, ^2^Purdue Institute for Cancer Research, ^3^Department of Industrial and Physical Pharmacy, Purdue University, ^4^Department of Biochemistry, Purdue University, ^5^Department of Comparative Pathobiology, Purdue University, ^6^Department of Computer Science, Purdue University, West Lafayette, IN 47907, USA.

*Corresponding author: [andrisao@purdue.edu](mailto:andrisao@purdue.edu)

Department of Basic Medical Sciences,

Purdue University

201 S. University Street

West Lafayette, IN  47907-2064

Phone: 765-494-8131

**Supplementary Figures**

**
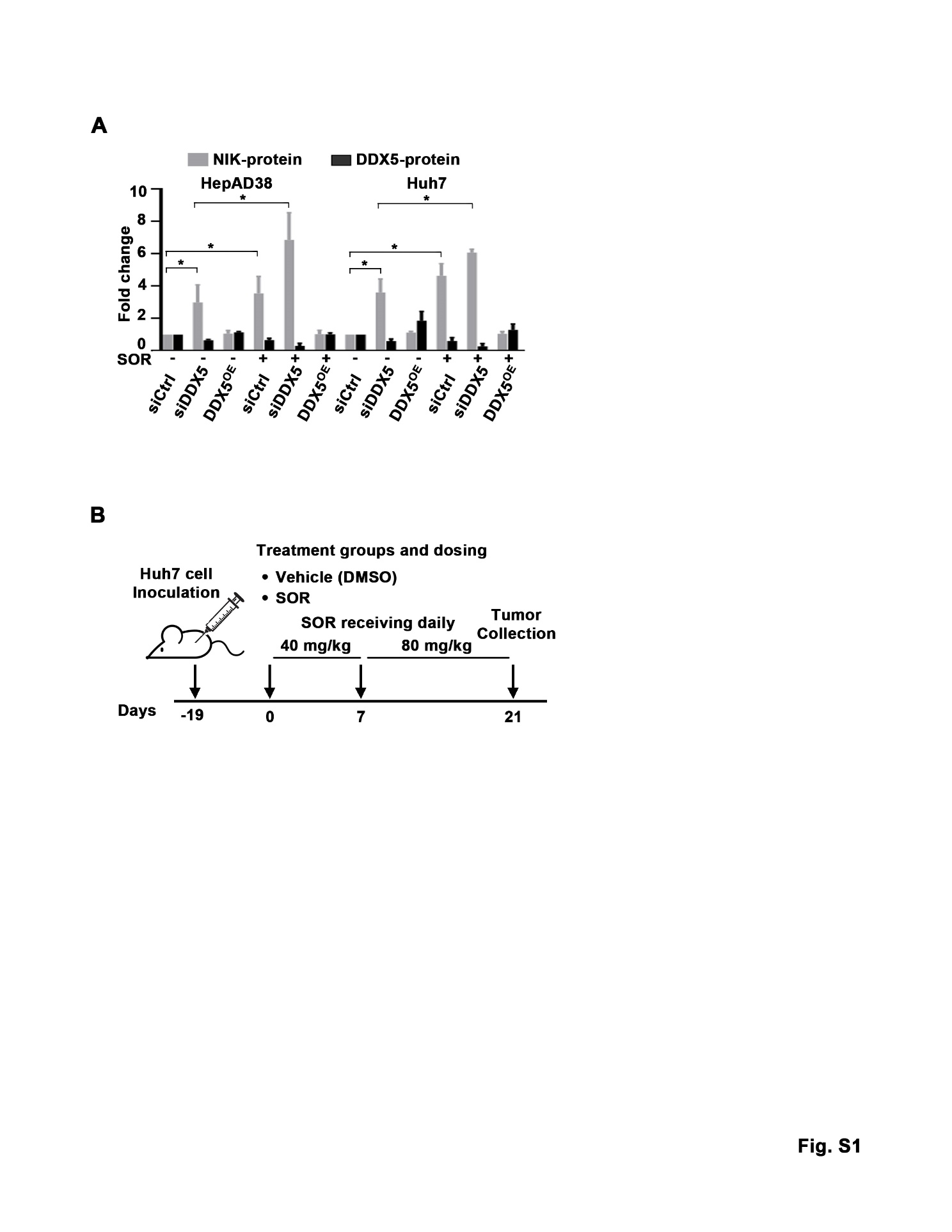
**

**Figure S1**. (**A)** Quantification by imageJ software of immunoblots of NIK using lysates from Dox-inducible-DDX5-Huh7 and -HepAD38 cell lines, transfected with siCtrl or siDDX5 as described in Fig.1D, +/- 10 µM SOR for 24h. For DDX5^OE^, Dox-inducible-DDX5 cell lines grown with Dox (1µg/ml) for 48 h and SOR for the last 24h. Error bars of immunoblot quantification data represent SD. *p<0.05, by unpaired *t-*test. Actin used as loading control. **(B)** Diagram illustrates treatment groups and timetable of SOR administration as described (7).

**
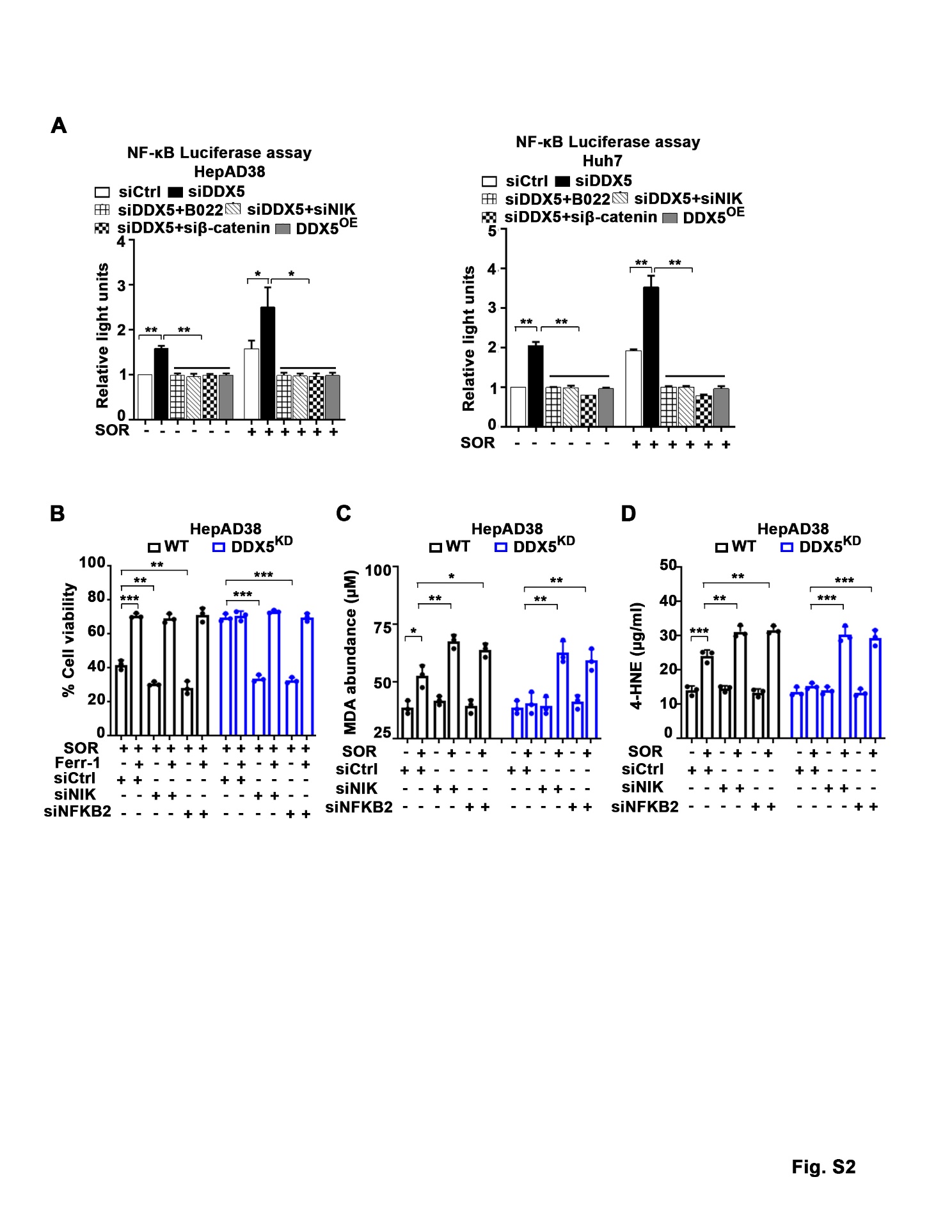
**

**Figure S2. (A)** NF-κB-response element (RE) luciferase vector (pNL3.2.NF-κB-RE vector, Promega) (1.0 µg per 12-well plate) co-transfected in HepAD38 and Huh7 cells with siCtrl or siDDX5, +/- SOR (10 µM), B022 (5.0 µM), siNIK (50 pM) or siβ-catenin (50 pM), as indicated, or in DDX5 ^OE^ cells, using the Dox-inducible-DDX5-HepAD38 cell line treated with Dox (1.0 µg/ml ) +/- SOR (10 µM) for 48h. Data are expressed as mean ± SEM from three independent experiments. *p<0.05, **p<0.01 by unpaired *t-*test. **(B)** Cell viability of HepAD38 WT or DDX5^KD^cells transfected with indicated siRNAs (50 pM each) treated with SOR (10 µM) +/- 10 Ferr-1 (10 µM) for 24h. Data expressed as mean ± SEM from n=3. **p<0.01, ***p<0.001 by unpaired *t-*test. **(C)** MDA and **(D)** 4-HNE abundance quantified using lysates from WT and DDX5^KD^ HepAD38 cells, transfected with indicated siRNAs (50 pM each), treated with SOR (10 µM) +/- 10 Ferr-1 (10 µM) for 24h. Data expressed as SD, n=3. *p<0.05, **p<0.01, ***p<0.001 by unpaired *t-*test.

**
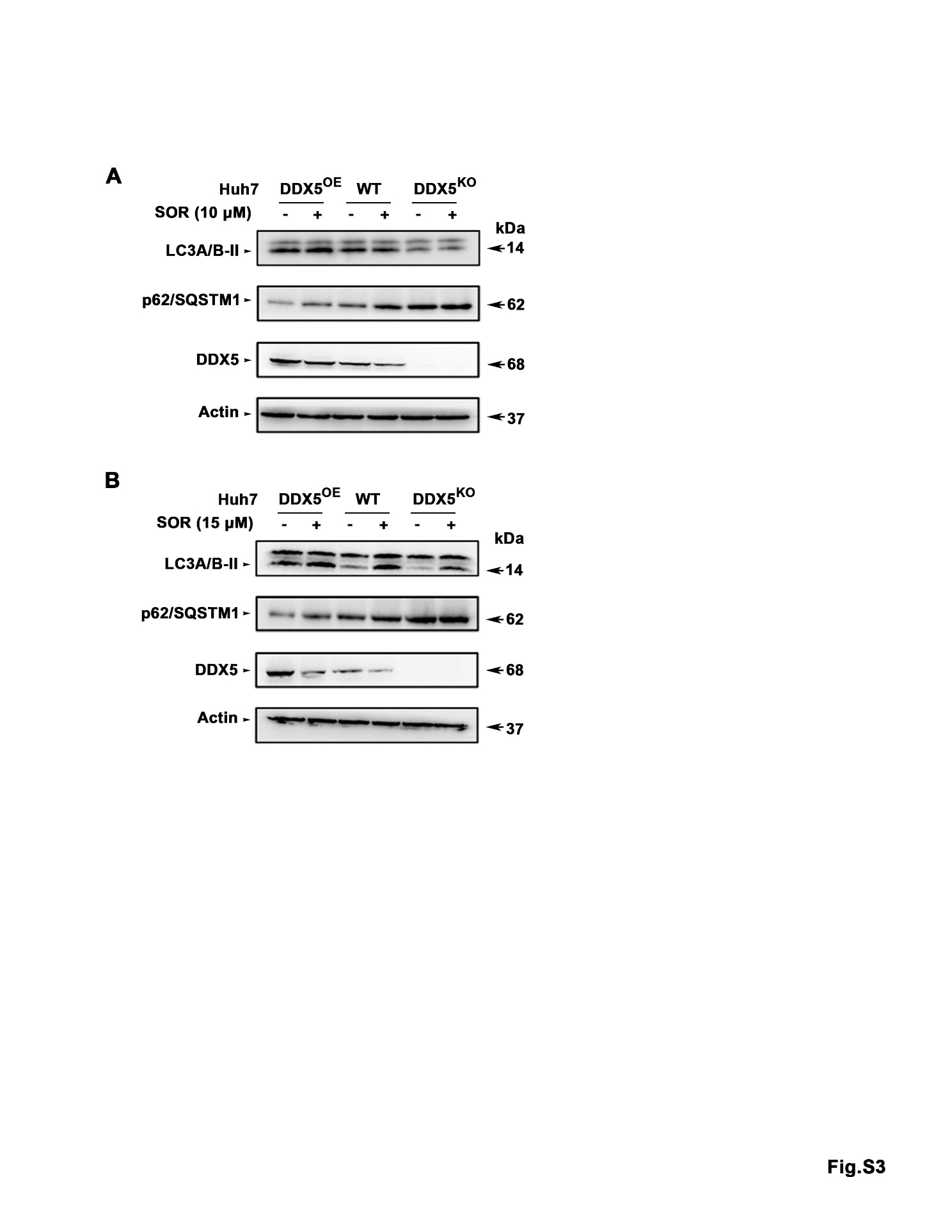
**

**Figure S3.** Immunoblots of LC3A/B, p62/SQSTM1 and DDX5 using lysates from Huh7 cells with DDX5 overexpression (DDX5^OE^) as described (7), WT DDX5, and DDX5^KO,^ treated with (+) or without (-) 10µM SOR for 24hr. A representative immunoblot is shown from n=3. Actin is used as loading control.

**
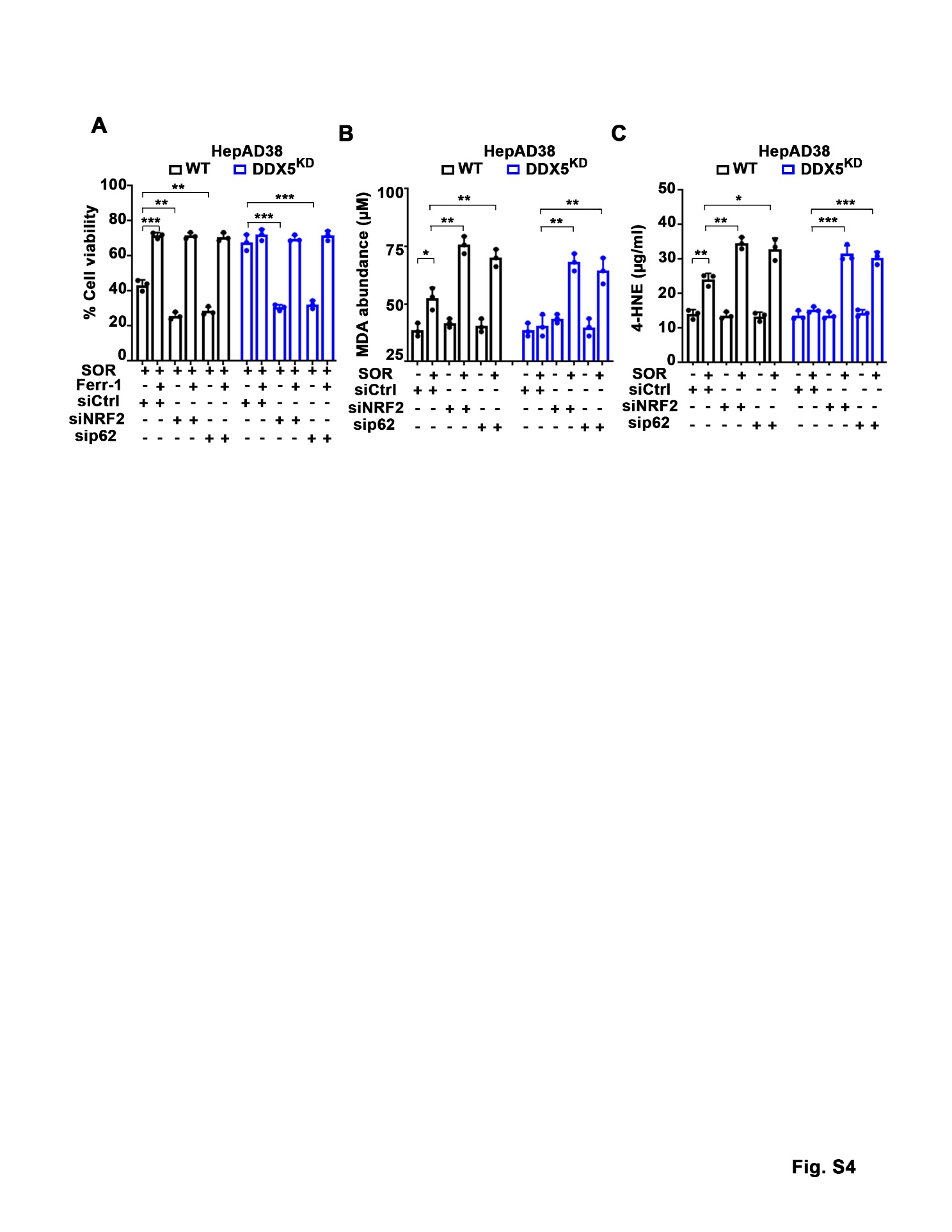
Figure S4: (A)** Cell viability of HepAD38 WT or DDX5^KD^cells transfected with indicated siRNAs (50 pM each) treated with SOR (10 µM) +/- 10 Ferr-1 (10 µM) for 24h. Data expressed as mean ± SEM from n=3. **p<0.01, ***p<0.001 by unpaired *t-*test. **(C)** MDA and **(D)** 4-HNE abundance quantified using lysates from HepAD38 WT and DDX5^KD^ cells, transfected with indicated siRNAs (50 pM each), treated with SOR (10 µM) +/- 10 Ferr-1 (10 µM) for 24h. Data expressed as SD, n=3. *p<0.05, **p<0.01, ***p<0.001 by unpaired *t-*test.


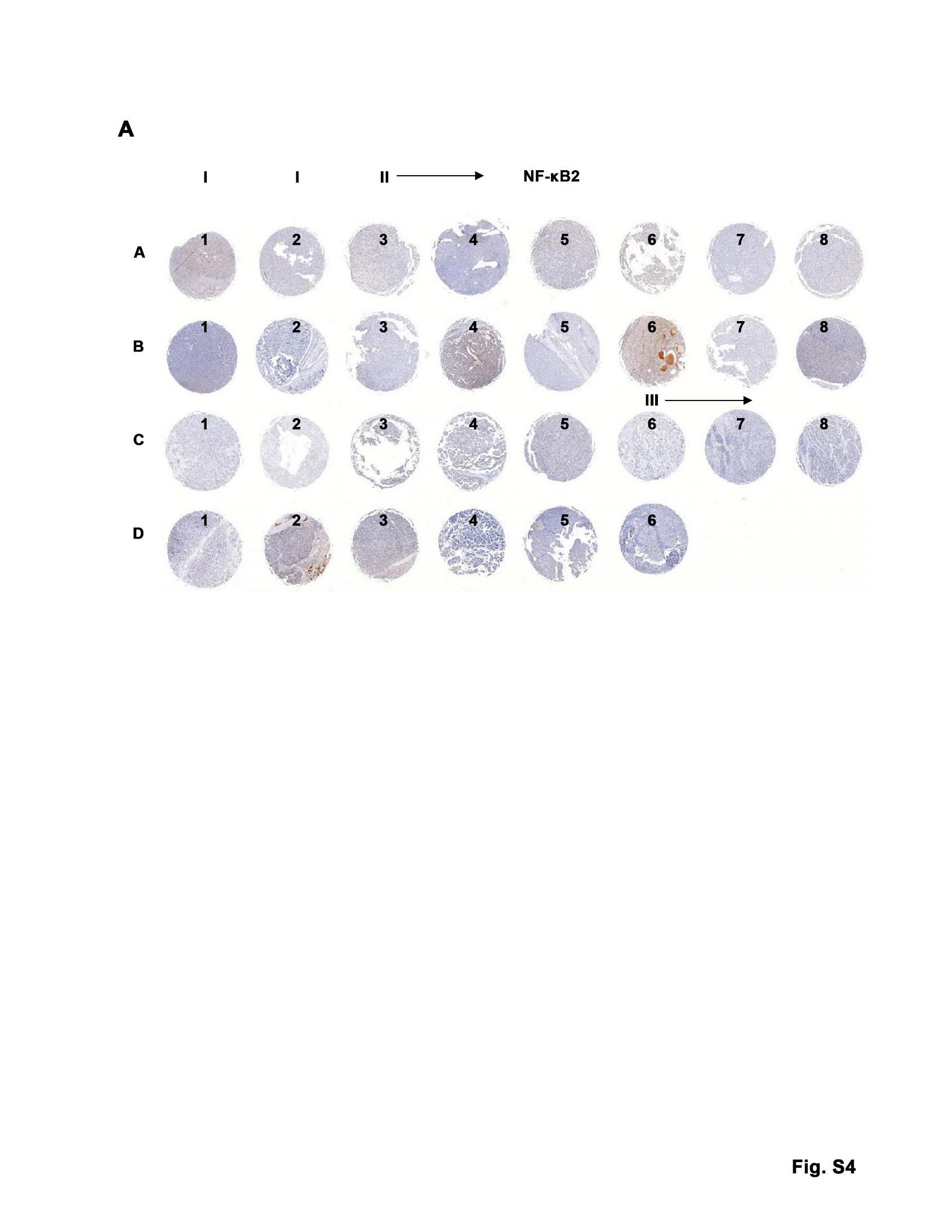


**Figure S5:** **(A)** Immunohistochemistry (IHC) with NFKB2/p52 antibody of tissue microarray (TMA) comprised of 30 human HCCs grades I-III. Images shown at 0.4X magnification. The same HCC TMA was used for IHC with DDX5 antibody, described in reference#7, by Li et al.


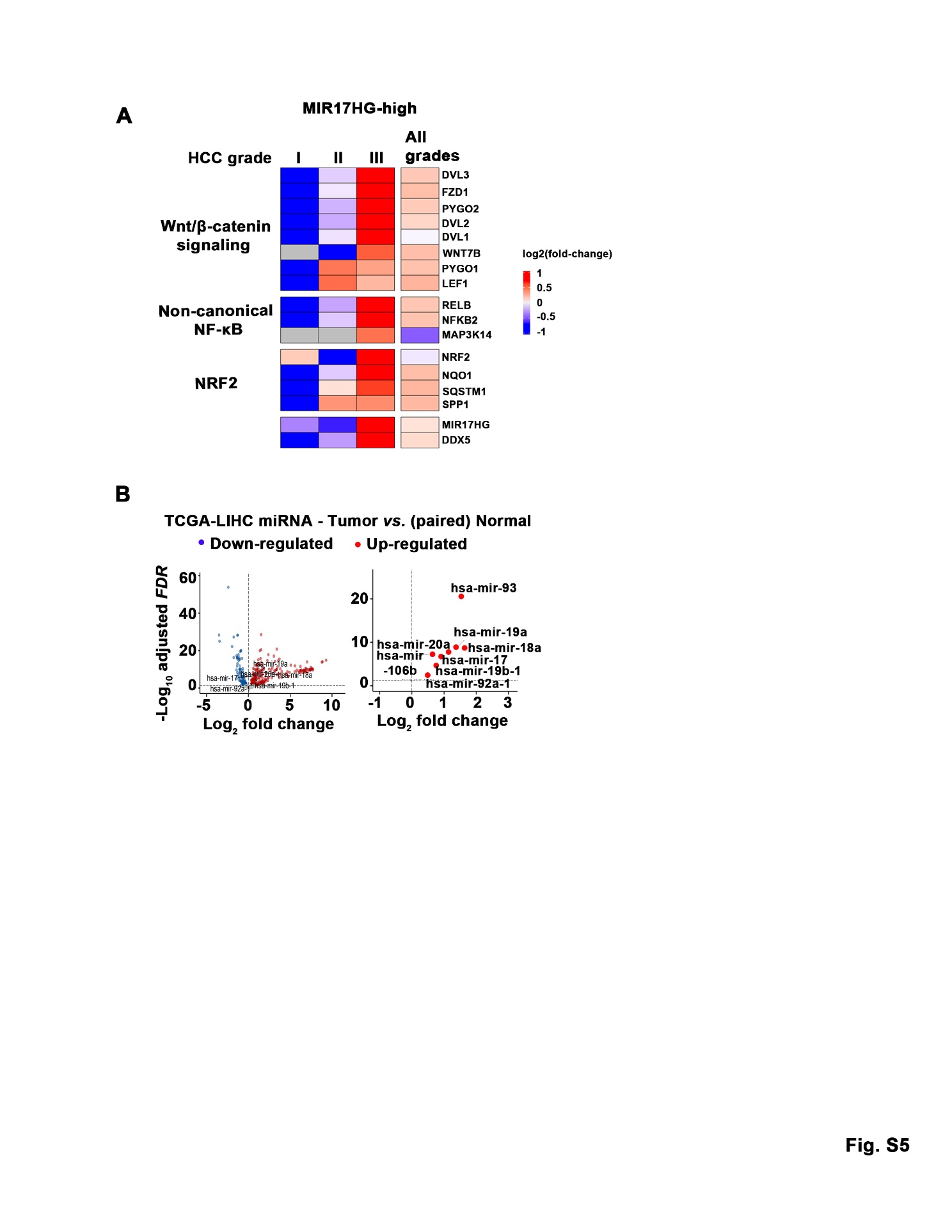


**Figure S6. (A)** Heatmap of MIR17HG-high across grades I-III HCC and indicated genes from Wnt/β-catenin signaling, non-canonical NF-kB, and NRF2 pathways. The average log2 fold-change expression values (scaled by row, FDR<0.2) comparing All HCC grades [n=185], grade I [n=30], grade II [n=78], and grade III [n=68] with paired normal [n=50] in TCGA-LIHC patients are displayed**. (B)** The miR17-92 cluster including the paralogue miR106b-25 cluster target various tumor suppressors including DDX5 (1).The volcano plot (right) shows the upregulated expression of these microRNAs in paired tumors vs normal liver.

**Supplementary Materials and Methods**

**Immunoblotting:** Cells were lysed (15 min, 4^o^C) in lysis buffer (Cell Signaling), sonicated on ice for 30 sec and clarified by centrifugation (13,000 rpm, 15 min, 4^o^ C). Protein concentration was determined using BCA assay. All samples were diluted to 1µg/µL using 4x dye (Biorad) and equal amounts of proteins (5.0 – 40 μg per lane) were run on SDS-PAGE. Following electrophoresis, proteins transferred to nitrocellulose membrane via wet transfer (200 mA, 45 - 90 min at 4^o^ C). Following transfer, membranes were blocked with 3% (w/v) BSA in Tris-buffered saline containing 0.1% (v/v) Tween 20 (TBST) and incubated with primary antibody in 3% (w/v) BSA in TBST for 1hr at room temperature, followed by incubation in secondary antibody (1:2000 dilution) in 3% BSA in TBST for 1h at room temperature. Three washes performed after primary and secondary antibody incubations. Protein bands detected by chemiluminescence using Pierce ECL (Biorad). Densitometric analysis of immunoblots performed using ImageJ software. Immunoblots are representative of three independent experiments. Antibodies used are listed in Supplementary Table S2:

**RNA preparation and qRT-PCR:** RNA was isolated using Purelink mRNA Mini kit (Life Technologies) or Direct Zol RNA miniprep kit (Zymo Research). cDNA synthesized from 1.0 μg total RNA using iSCRIPT cDNA synthesis kit (Biorad). qRT-PCR performed using SYBR green (Roche) in triplicates, normalized to GAPDH.

**Chromatin Immunoprecipitation (ChIP):** ChIP assays were performed using the Pierce Agarose ChIP Kit (Thermo Fisher). HepAD38 WT and DDX5^KD^ cells were cross-linked with formaldehyde to a final concentration of 1%. Approximately 2x10^6^ cells were used per ChIP assay. Immunoprecipitations were carried out with the indicated antibodies (Supporting Table S2). After reversing the cross-linking, DNA was extracted and subjected to real-time quantitative PCR (qPCR) using the corresponding primers (Supporting Table S3).

**Nanosac preparation:** Nanosac carrying siCtrl, siβ-catenin, and siNRF2 prepared as previously described (2). Nanosac-encapsulated siRNAs were administered every 48h intra-tumorally, delivering 3µg siRNA per injection. Briefly, a sacrificial core of Nanosac, mesoporous silica nanoparticle (MSN), was prepared by adding a silica precursor, tetraethyl orthosilicate (TEOS), and -triethanolamine to micelles of cetyltrimethylammonium chloride (CTAC). Once silica layers formed on the micelles, the surfactant was removed by washing MSNs with methanol and HCl (500:19, v/v) for 48 h at room temperature. MSNs were collected by centrifugation at 17000 Relative Centrifugal Force (rcf) for 15 min, washed three times with methanol and cationized by 3-aminopropyltriethoxysilane (APTES). Amine-modified MSN-APTES (MSN^a^) particles were collected by centrifugation at 17000 rcf for 15 min and washed three time with ethanol. The purified MSN^a^ was mixed with siRNA (siCtrl, or siNRF2) at a weight ratio of 150/1 (MSN^a^/siRNA) and incubated for 5 min at room temperature. The siRNA-loaded MSNs were incubated with dopamine hydrochloride solution in Tris buffer (10 mM, pH 8.5) for 24 hours at room temperature for polydopamine (pD) surface coating. After removing the remaining dopamine, the pD-coated siRNA-MSNs were mixed with an etch solution (0.36 M HF/1.44 M NH4F, pH 5) for 5 min to remove the sacrificial MSN core, washed three times with deionized water, and collected by centrifugation at 300 rcf for 10 min.

**Analysis of TCGA.LIHC and other data RNAseq:** TCGA-LIHC RNAseq data (raw and TPM counts) for Tumor (n=371) and paired-normal (n=50) was downloaded using R-package TCGAbiolinks (3). Metadata for TCGA-LIHC patients was downloaded from cBioPortal (4) accessed in February 2024). TCGA patients (tumor) were grouped into 4 quantiles based on TPM expression value for gene MIR17HG. Patients stratified into top two quantiles (n=185) were denoted as “MIR17HG.high” group. RNAseq data for STAM and chow-diet fed mice were downloaded from NCBI (Accession: SRP159121 and GSE199092), respectively. Differentially expressed genes between (i) MIR17HG.high vs. paired-normal; (ii) MIR17HG.high (grade I) vs. paired-normal; (iii) MIR17HG.high (grade II) vs. paired-normal; and (vi) MIR17HG.high (grade III) vs. paired-normal. STAM vs. chow-diet fed mice were determined using edgeR(5) and (Supplementary Table S1). Data visualizations were performed using R-packages ComplexHeatmap (6) ggplot2 (7) and ggpubr (8).

**References**

1. Mani SKK, Yan B, Cui Z, Sun J, Utturkar S, Foca A, et al. Restoration of RNA helicase DDX5 suppresses hepatitis B virus (HBV) biosynthesis and Wnt signaling in HBV-related hepatocellular carcinoma. Theranostics 2020;10:10957-10972.

2. Kim H, Yuk SA, Dieterly AM, Kwon S, Park J, Meng F, et al. Nanosac, a Noncationic and Soft Polyphenol Nanocapsule, Enables Systemic Delivery of siRNA to Solid Tumors. ACS Nano 2021;15:4576-4593.

3. Colaprico A, Silva TC, Olsen C, Garofano L, Cava C, Garolini D, et al. TCGAbiolinks: an R/Bioconductor package for integrative analysis of TCGA data. Nucleic Acids Res 2016;44:e71.

4. de Bruijn I, Kundra R, Mastrogiacomo B, Tran TN, Sikina L, Mazor T, et al. Analysis and Visualization of Longitudinal Genomic and Clinical Data from the AACR Project GENIE Biopharma Collaborative in cBioPortal. Cancer Res 2023;83:3861-3867.

5. Robinson MD, McCarthy DJ, Smyth GK. edgeR: a Bioconductor package for differential expression analysis of digital gene expression data. Bioinformatics 2010;26:139-140.

6. Gu Z, Eils R, Schlesner M. Complex heatmaps reveal patterns and correlations in multidimensional genomic data. Bioinformatics 2016;32:2847-2849.

7. Wickham H (2016). ggplot2: Elegant Graphics for Data Analysis. Springer-Verlag New York. ISBN 978-3-319-24277-4, [https://ggplot2.tidyverse.org](https://ggplot2.tidyverse.org/).

8. Kassambara A (2023). *ggpubr: 'ggplot2' Based Publication Ready Plots*. R package version 0.6.0, <https://rpkgs.datanovia.com/ggpubr/>.

**Supplementary Table S1: List of Plasmids and siRNAs**

| **Plasmids, siRNAs** | **Source** |
| --- | --- |
| Renilla luciferase vector | Addgene (#27163) |
| TOPFlash vector | Addgene (#12456) |
| NanoLuc® Reporter Vector with NF-κB Response Element (RE) | Promega (# N1111) |
| MAP3K14-Luciferase vector | Kindly provided by Dr. Inoue, J. (ref. 29) |
| siCtrl | ThermoFisher Scientific (#4390843) |
| siDDX5-1 | ThermoFisher Scientific (#4392420, assay id s4007) |
| siDDX5-2 | ThermoFisher Scientific (#4392420, assay id s4008) |
| siNRF2 | ThermoFisher Scientific (#107966, assay id AM16708) |
| siNIK | ThermoFisher Scientific (#110821, assay id AM16708) |
| siCTNNB1(catenin beta 1) | ThermoFisher Scientific (#146154, assay id AM16708) |
| siNFKB2 | ThermoFisher Scientific (#106835, assay id AM16708) |
| siSQSTM1/p62 | Horizon (SMARTpool, L-010230-00-0005) |

**Supplementary Table S2: Antibodies Used**

| **Antibody** | **Dilution** | **Application** | **Source** |
| --- | --- | --- | --- |
| Mouse α-Human DDX5 | 1:1000 in 2% BSA in TBST | Western Blot | Millipore Sigma (#05-850) |
| Rabbit α-Human DDX5 | 1:1000 in 2% BSA in TBST | Western Blot | Cell Signaling Technologies (#14994S) |
| Mouse α-Human KEAP1 | 1:1000 in 2% BSA in TBST | Western Blot | Santa Cruz Biotechnology (#sc-365626) |
| Rabbit α-Human SQSTM1/p62 | 1:1000 in 2% BSA in TBST | Western Blot | Cell Signaling Technologies (#5114S) |
| Rabbit α-Human NRF2 | 1:1000 in 2% BSA in TBST | Western Blot | Cell Signaling Technologies (#12721S) |
| Rabbit α-Human NF-κB2 p100/p52 | 1:1000 in 2% BSA in TBST | Western Blot | Cell Signaling Technologies (#4882S) |
| Rabbit α-Human NF-κB2 p100/p52 | 1/50 | IHC | Proteintech  (#10409-2-AP) |
| Mouse α-Human RelB | 1:1000 in 2% BSA in TBST | Western Blot | Santa Cruz Biotechnology (#sc-48366) |
| Mouse α-Human NFκB p65 | 1:1000 in 2% BSA in TBST | Western Blot | Santa Cruz Biotechnology (#sc-8008) |
| Rabbit α-Human SUZ12 | 1:1000 in 2% BSA in TBST | Western Blot | Cell Signaling Technologies (#3737S) |
| Mouse α-Human Actin | 1:1000 in 2% BSA in TBST | Western Blot | Sigma (#A5441) |
| Horse α-Mouse secondary | 1:2000 in 2% BSA in TBST | Western Blot | Vector Laboratories (#PI-2000) |
| Goat α-Rabbit secondary | 1:2000 in 2% BSA in TBST | Western Blot | Vector Laboratories (#PI-1000) |
| Mouse FLAG | 1:1000 in 2% BSA in TBST | Western Blot | Sigma (#F1804) |
| NIK | 1:1000 in 2% BSA in TBST | Western Blot | Cell Signaling Technologies (#4994S) |
| Pol II ChIP validated | 10μl per each IP | IP | ThermoFisher Scientific (#26156) |
| H3 ChIP validated | 10μl per each IP | IP | ThermoFisher Scientific (#701517) |
| H3K27me3 ChIP validated | 10μl per each IP | IP | Abcam (#ab6002) |
| P52-ChIP validated | 10μl per each IP | IP | Cell Signaling Technologies (#37359S) |
| Rabbit α-DDX5 | 1/1000 | IHC | Abcam (#ab21696) |
| Histone H3 | 1/1000 | Western Blot | Cell Signaling Technologies (#4620S) |
| LC3A/B | 1/1000 | Western Blot | Cell Signaling Technologies (#12741S) |

**Supplementary Table S3: Primer sequences**

| **Primer** | **5’ – Sequence – 3’** |
| --- | --- |
| DDX5-F | AGCAAGTGAGCGACCTTATC |
| DDX5-R | CATCCTTCATGCCTCCTCTAC |
| GAPDH-F | CCCTTCATTGACCTCAACTACA |
| GAPDH-R | ATGACAAGCTTCCCGTTCTC |
| UBc-F | CCTGGAGGAGAAGAGGAAAGAGA |
| UBc-R | TTGAGGACCTCTGTGTATTTGTCA |
| NRF2-F | TTCAGATGCCACAGTCAACAC |
| NRF2-R | GGCATGCTGTTGCTGATACT |
| β-catenin-F | CACAAGCAGAGTGCTGAAGGTG |
| β-catenin-R | GATTCCTGAGAGTCCAAAGACAG |
| NIK-F | TTGGTTGGGGAGATCGGCGCT TG |
| NIK-R | GGGGCTGAACTCTTGGCTATTCTC |
| mDDX5-F | ACGAATCTGTGGTCCTTTGG |
| mDDX5-R | CATAAACCACCAGCCATTCC |
| mGAPDH-F | GTCAAGGCCGAGAATGGGAA |
| mGAPDH-R | GCCTTCTCCATGGTGGTGAA |
| CHIP-NRF2-F | CTGTTGAAGGGCGCCATCTG |
| CHIP-NRF2-R | AAGCTCAGGTCGTCAAAGGC |

**Supplementary Table S4: Reagents, Chemical inhibitors, and Kits**

| **Reagents, Chemical inhibitors, Kits** | **Source** |
| --- | --- |
| Cycloheximide (CHX) | Selleck Chemicals (#S7418) |
| MG132 | Selleck Chemicals (#S2619) |
| Sorafenib | Selleck Chemicals (#S7397) |
| Ferrostatin | Selleck Chemicals (#S7243) |
| XAV-939 | Selleck Chemicals (#S1180) |
| CellTiter 96® AQueous One Solution Cell  Proliferation Assay (MTS) | Promega (#G3580) |
| PCR Mycoplasma Detection Kit | Abm (#G238) |
| Dual-Luciferase® Reporter Assay System | Promega (#1980) |
| Cell Lysis Buffer (10X) | Cell Signaling Technology (#9803) |
| LightCycler® 480 SYBR Green I Master | Roche (#04887352001) |
| iScript™ cDNA Synthesis Kit | Biorad (#1708891) |
| Nitrocellulose Membrane, Roll, 0.2 µm | Biorad (#1620112) |
| LightCycler® 480 Sealing Foil | Roche (#04729757001) |
| LightCycler® 8-Tube Strips (white) | Roche (#06612601001) |
| DMSO | Sigma (#D8418-50ML) |
| Tween™ 20 | ThermoFisher Scientific (BP337-500) |
| Triton™ X-100 | Sigma (#T8787-100ML) |
| Costar® 6-well Ultra-Low Attachment Plates | Corning (#3471) |
| Tetracycline hydrochloride | Sigma (#T7660-5G) |
| Bovine Serum Albumin | Sigma (#A9647-100G) |
| Pierce™ ECL Western Blotting Substrate | ThermoFisher Scientific (#32106) |
| Geneticin™ Selective Antibiotic (G418 Sulfate) | ThermoFisher Scientific (#10131027) |
| Pierce™ BCA Protein Assay Kit | ThermoFisher Scientific (#23227) |
| Lipofectamine™ 3000 Transfection Reagent | ThermoFisher Scientific (#L3000015) |
| Lipofectamine™ RNAiMAX Transfection Reagent | ThermoFisher Scientific (#13778150) |
| Restore™ PLUS Western Blot Stripping Buffer | ThermoFisher Scientific (#46430) |
| RNeasy Mini Kit | Qiagen (#74104) |
| Hoechst 33342 Solution (20 mM) | ThermoFisher Scientific (#62249) |
| Corning® 96 Well Black Polystyrene Microplate | Corning(#CLS3603-48EA) |
| CellTiter-Glo® 2.0 Cell Viability Assay | Promega (#G9242) |
| BODIPY™ 581/591 C11 (Lipid Peroxidation Sensor) | ThermoFisher Scientific (# D3861) |
| 29 mm Glass bottom dish with 14 mm micro-well #1.5 cover glass | NC0662883（D29-14-1.5-N） |
| Nano-Glo® Luciferase Assay System | Promega (# N1110) |
| RNAlater™ Stabilization Solution | ThermoFisher Scientific (# AM7024) |
| PEG400 | Selleck Chemicals (#S6705) |
| NE-PER™ Nuclear and Cytoplasmic Extraction Reagents | ThermoFisher Scientific (# 78835) |
| Pierce™ 16% Formaldehyde (w/v), Methanol-free | ThermoFisher Scientific (# 28908) |
| B022 | MedChemExpress (# HY-120501) |
| Protease Inhibitor Cocktail (100X) | Cell Signaling Technology (#5871) |
| NucRed™ Dead 647 ReadyProbes™ Reagent (TO-PRO-3 iodide) | ThermoFisher Scientific (# R37113) |
| Malondialdehyde (MDA) lipid peroxidation Assay Kit | Abcam (ab233471) |
| Lipid Peroxidation (4-HNE) Assay Kit (96 TESTS) | Abcam (ab238538) |
| Liver Hepatocellular carcinoma tissue microarray | US Biolab Corporation  (HLivH030PG020) |
|  |  |

**Supplementary Table S5**
