# Supplementary material for: DDX5 deficiency drives non-canonical NF-κB activation and NRF2 expression, influencing sorafenib response and hepatocellular carcinoma progression
Source: Cell Death Dis. 2024 Aug 9;15(8):583. doi: 10.1038/s41419-024-06977-z (PMC11315975; doi:10.1038/s41419-024-06977-z)
Supplement: Supplementary file 2 — Western Blots [file 41419_2024_6977_MOESM2_ESM.pdf]

Immunoblotting protocol is included under Supplementary Information section.

Please note: SDS PAGE analyses of lysates used for immunoblotting utilized prestained MW markers (Precision Plus Protein Dual Color Standards, #1610374, from BIO-RAD).

Following transfer, nitrocellulose membranes were cropped according to migration of prestained MW markers.

Fig1C

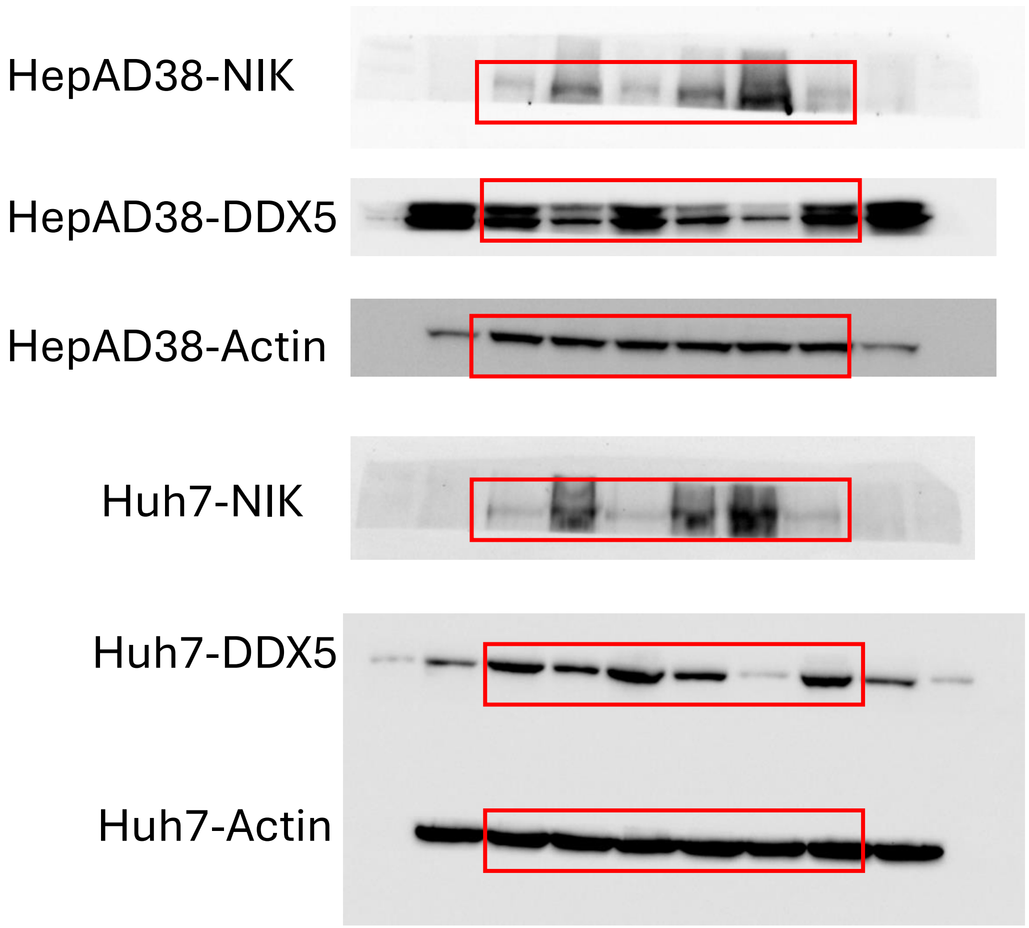
HepAD38-NIK
HepAD38-DDX5
HepAD38-Actin
Huh7-NIK
Huh7-DDX5
Huh7-Actin

Fig1F

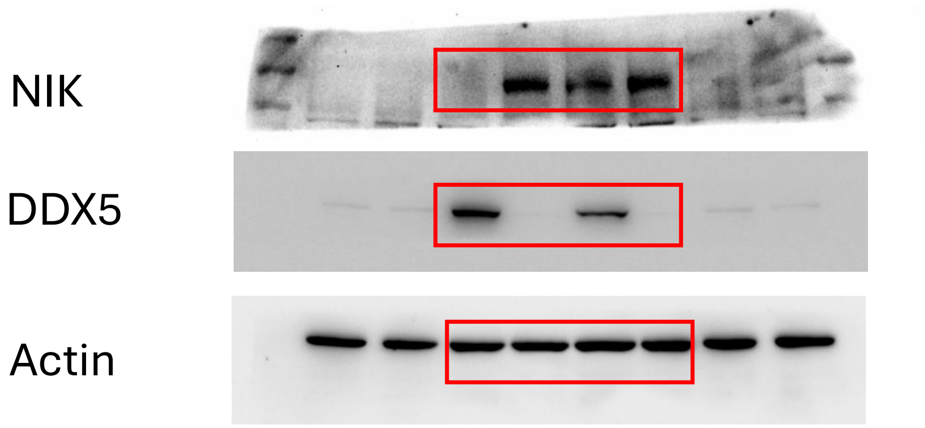

Fig3A

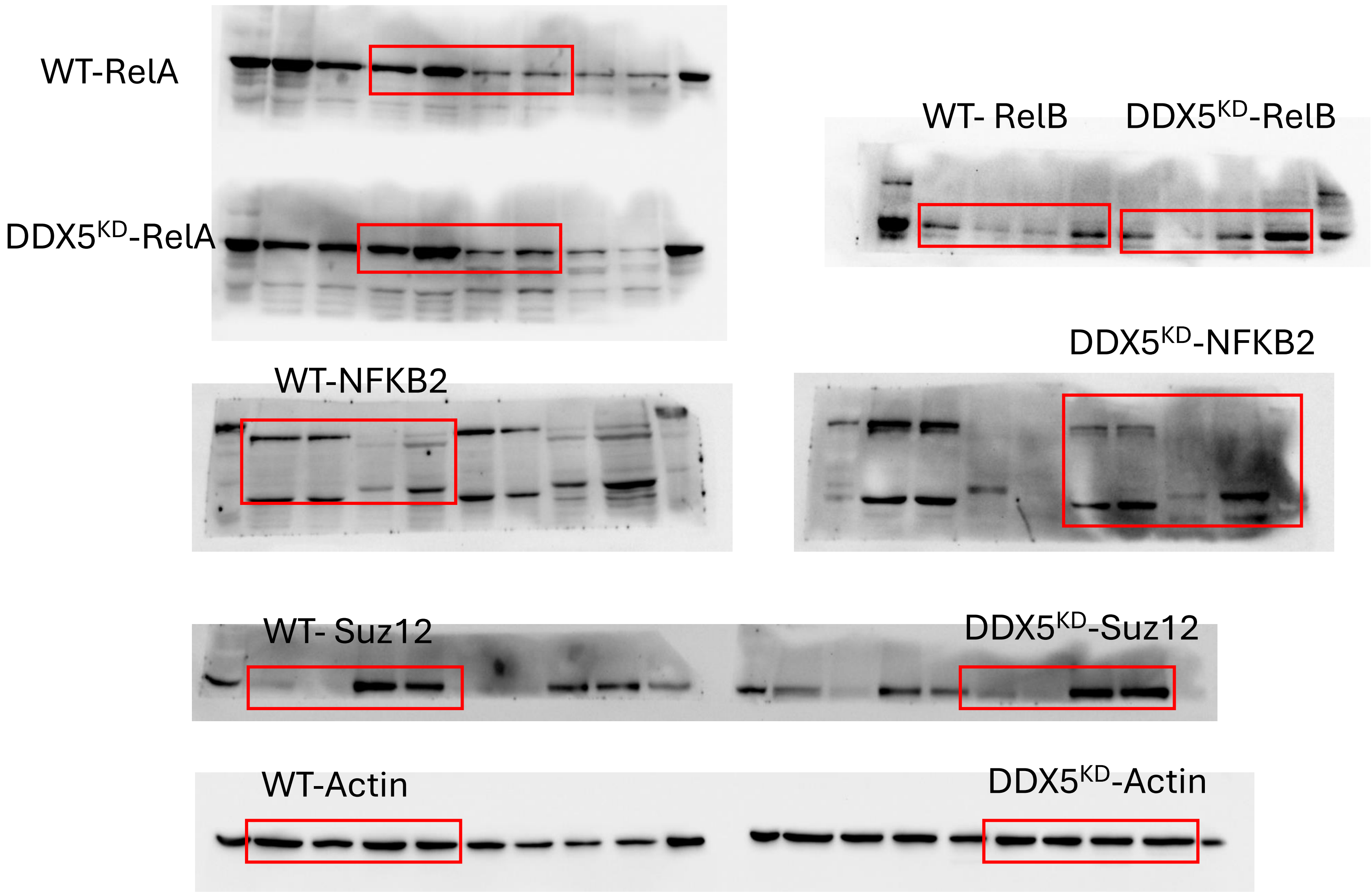
WT-RelA
DDX5^{KD}-RelA
WT- RelB
DDX5^{KD}-RelB
WT-NFKB2
DDX5^{KD}-NFKB2
WT- Suz12
DDX5^{KD}-Suz12
WT-Actin
DDX5^{KD}-Actin

Fig3B

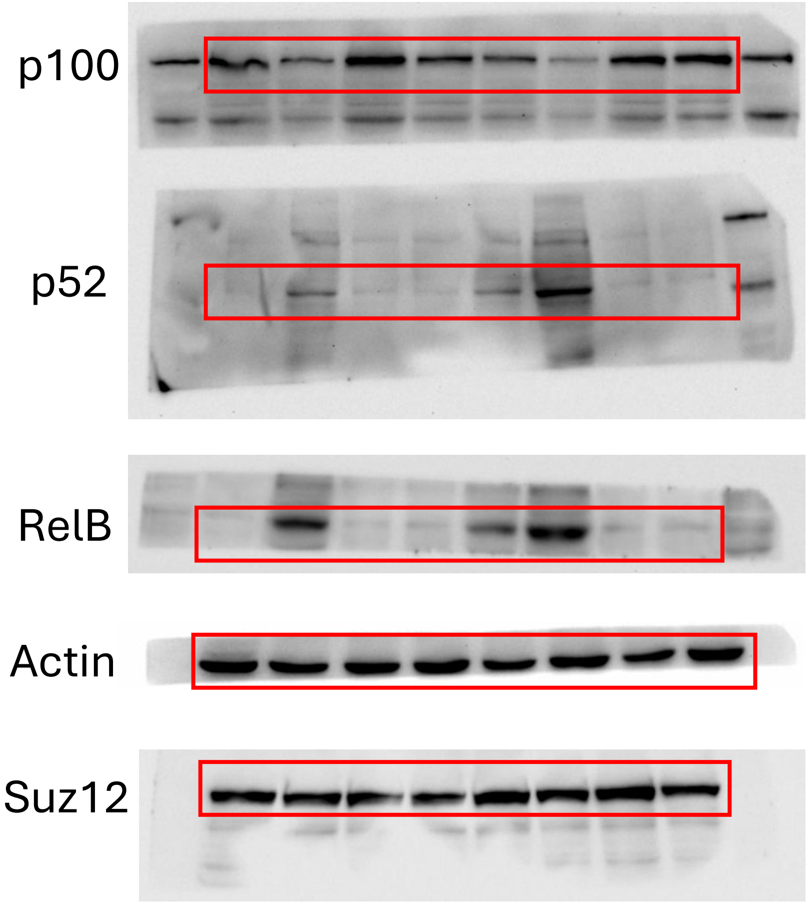

Fig3C

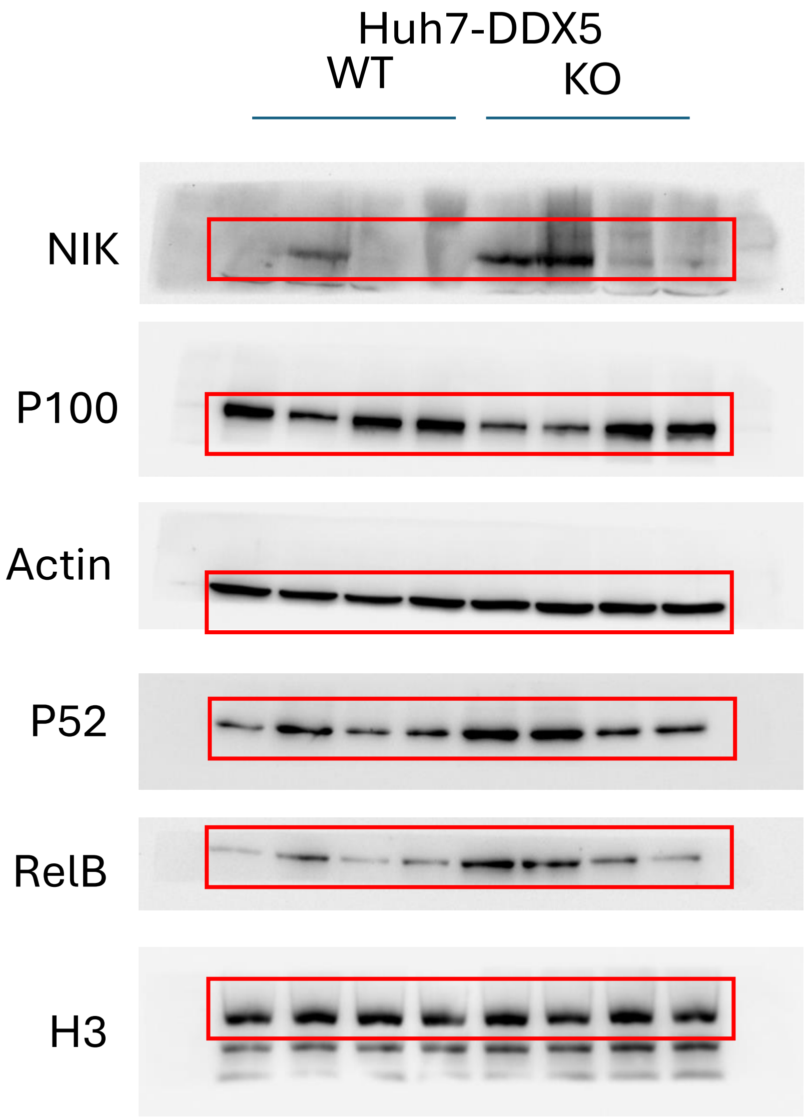

Fig4B

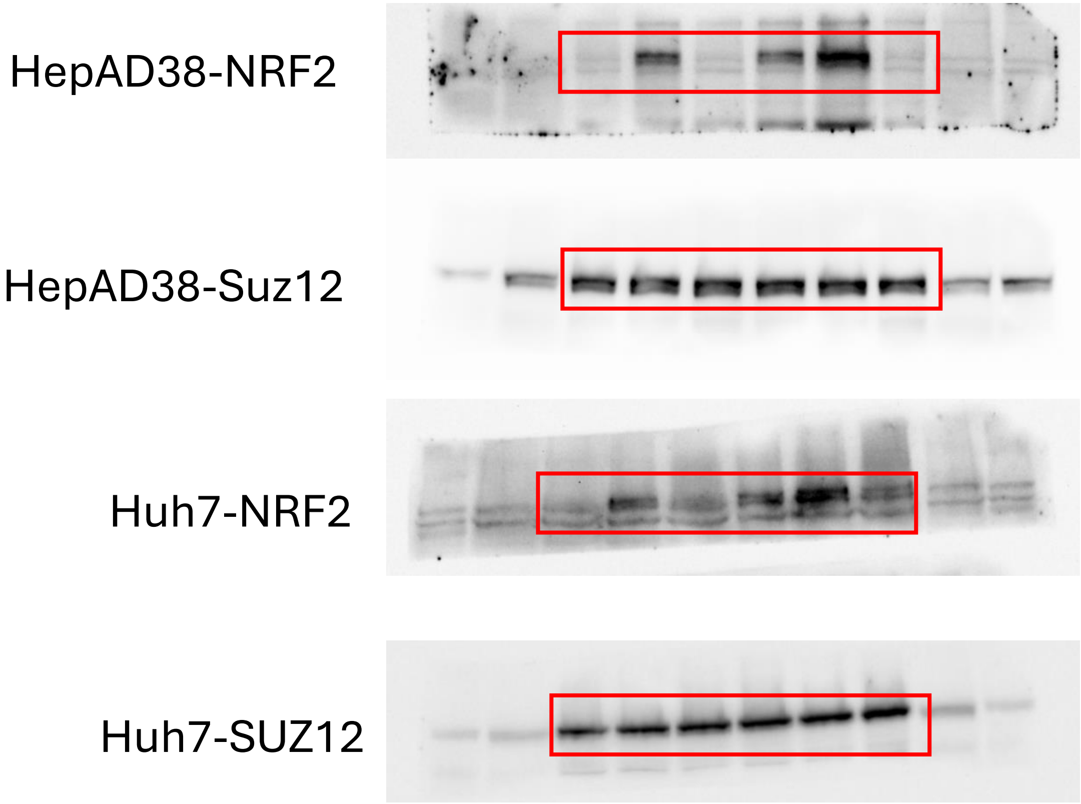

Fig4F

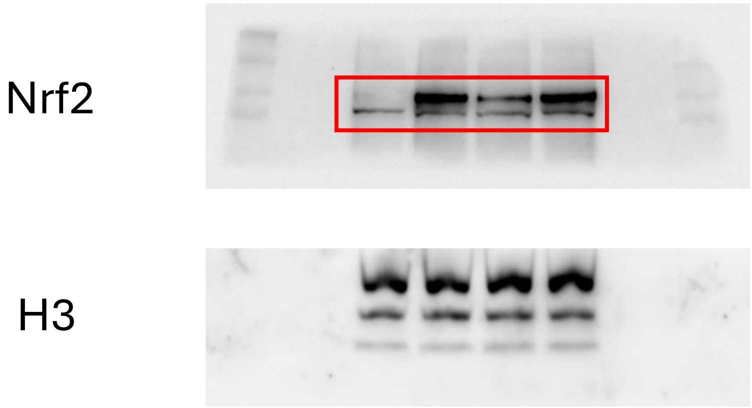

Fig5A

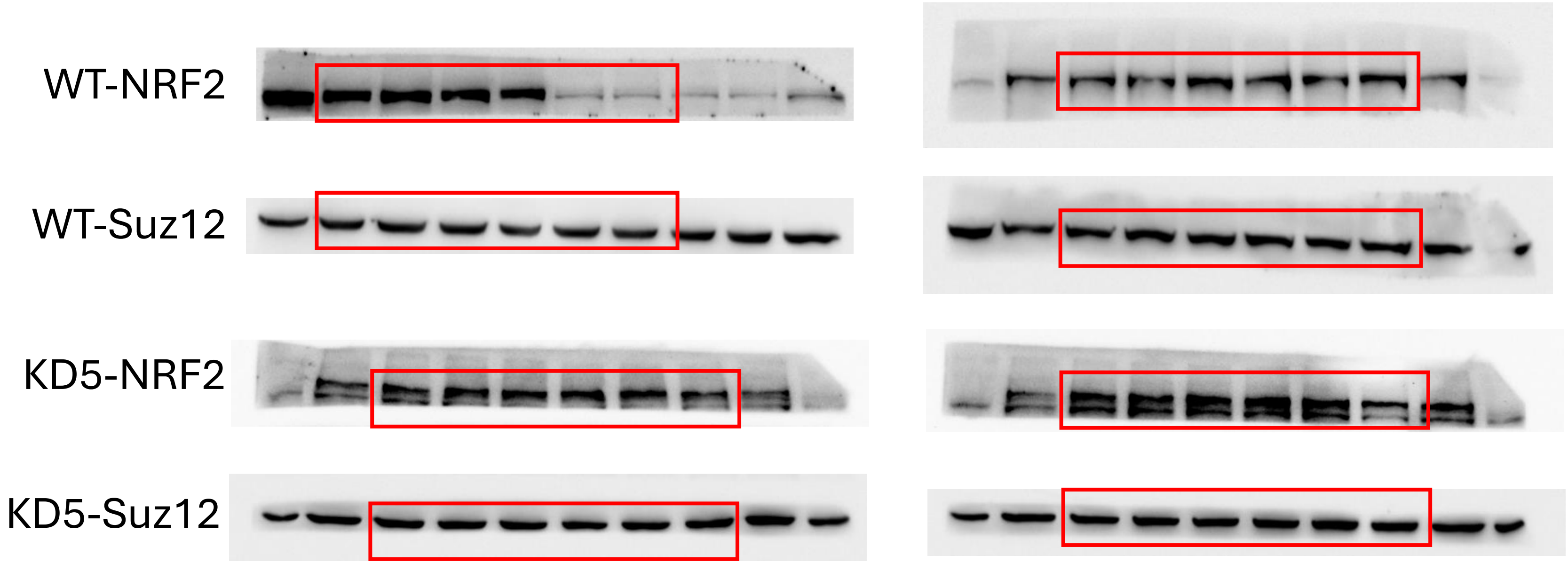

Fig5B

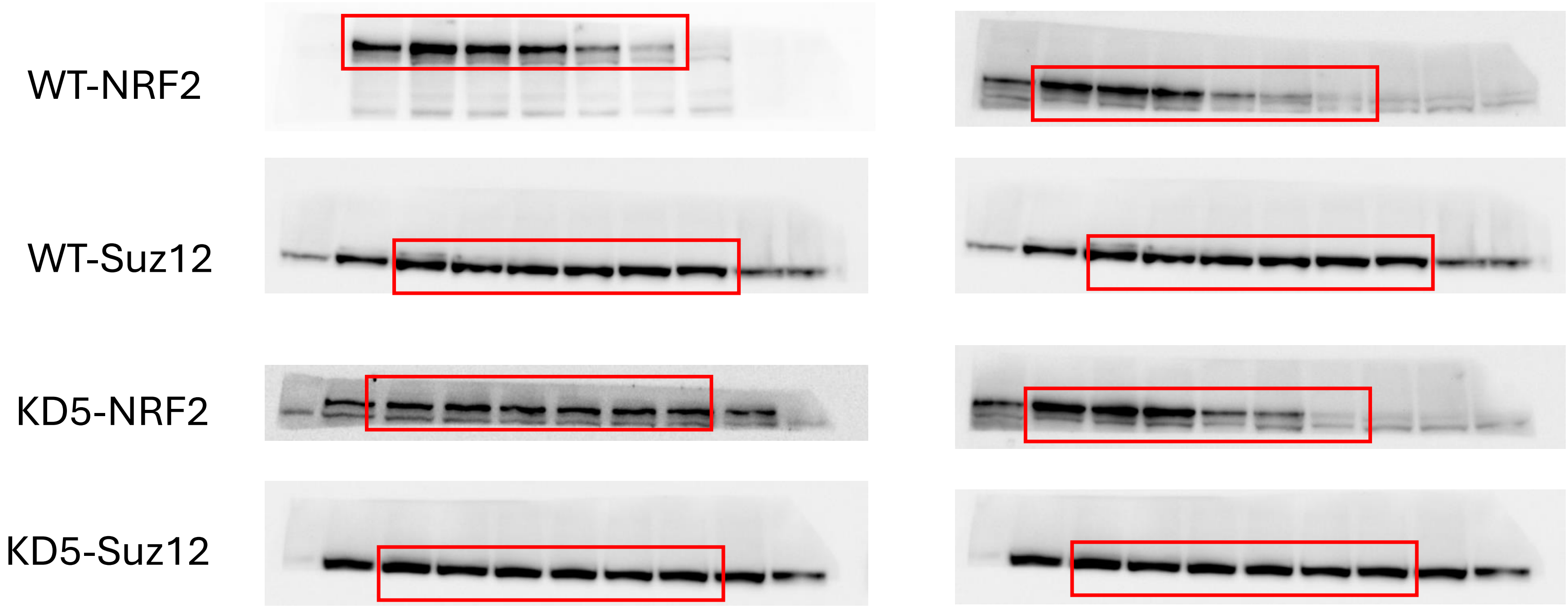

Fig5C

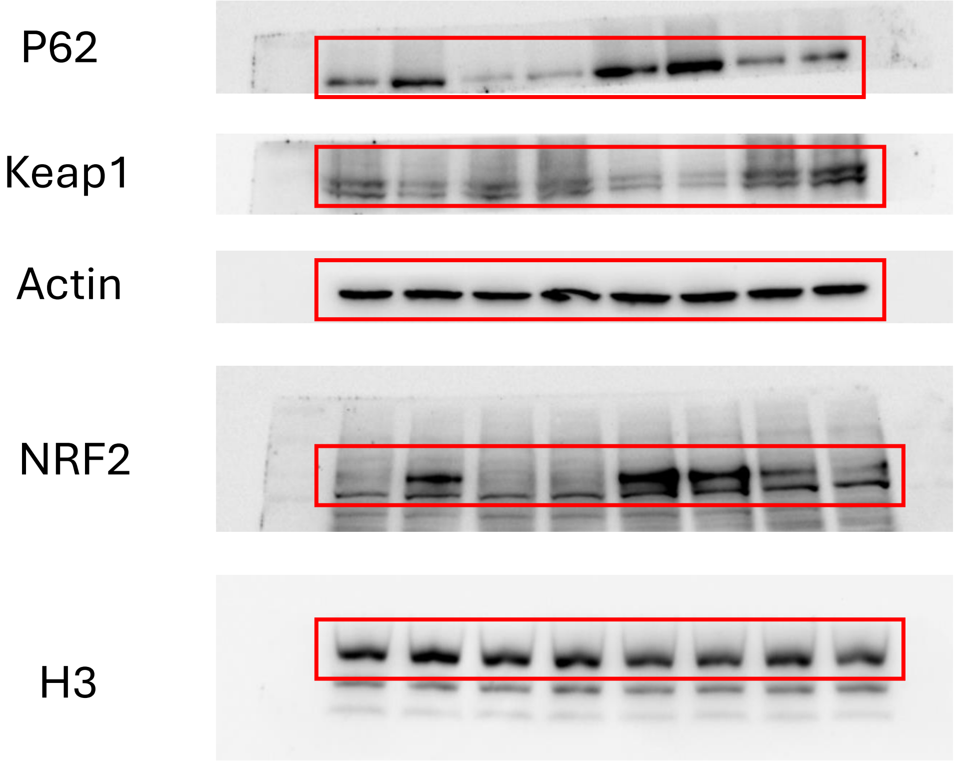

Fig6G

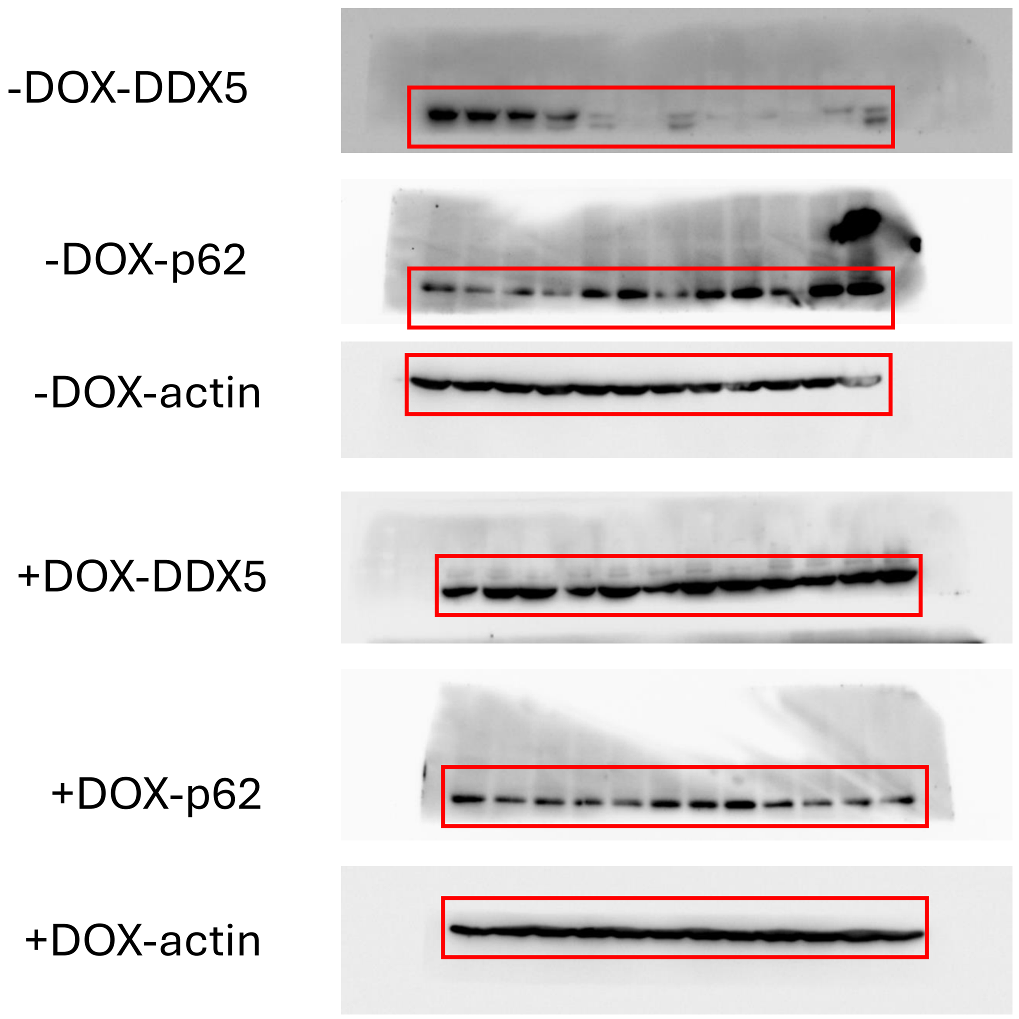

Fig7B

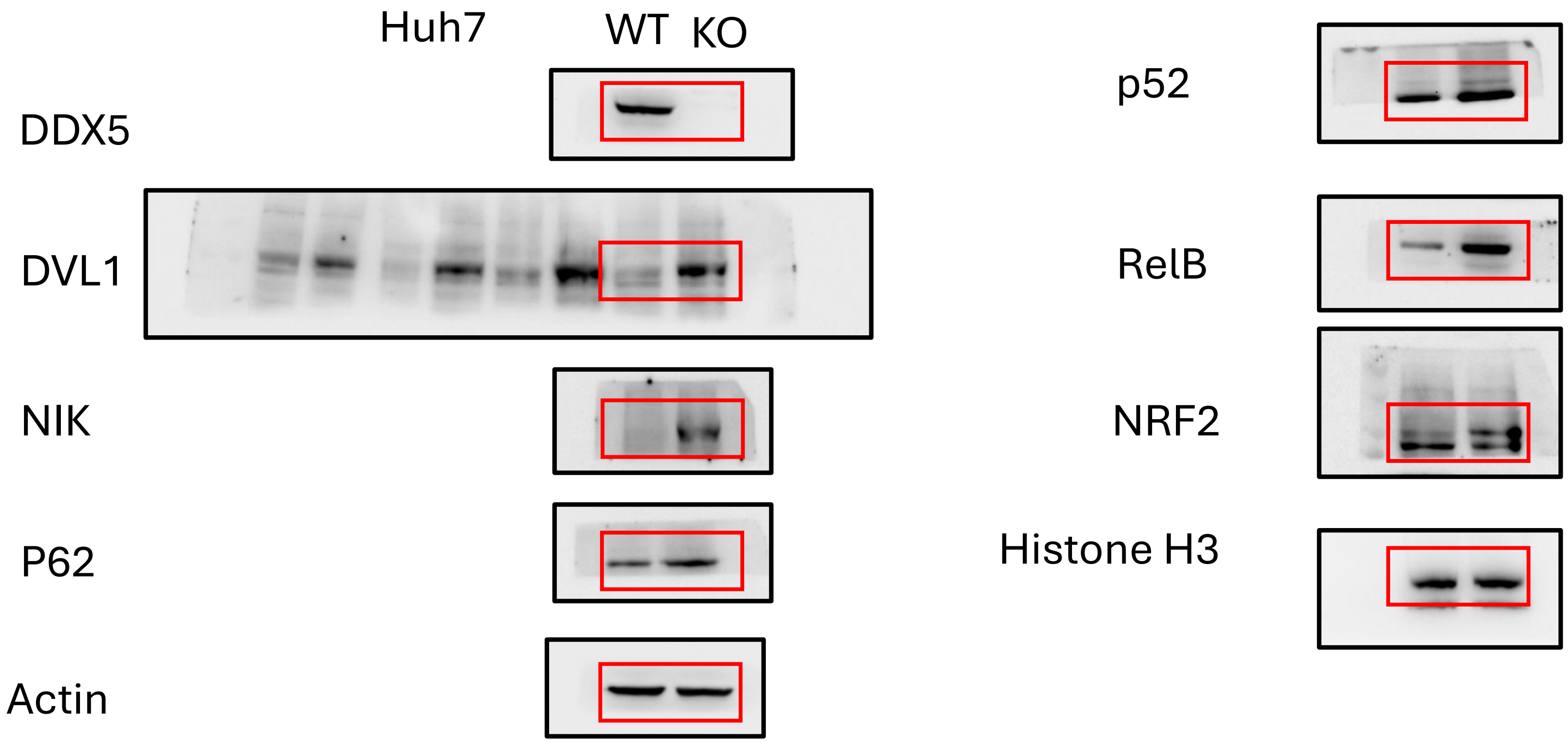

FigS3

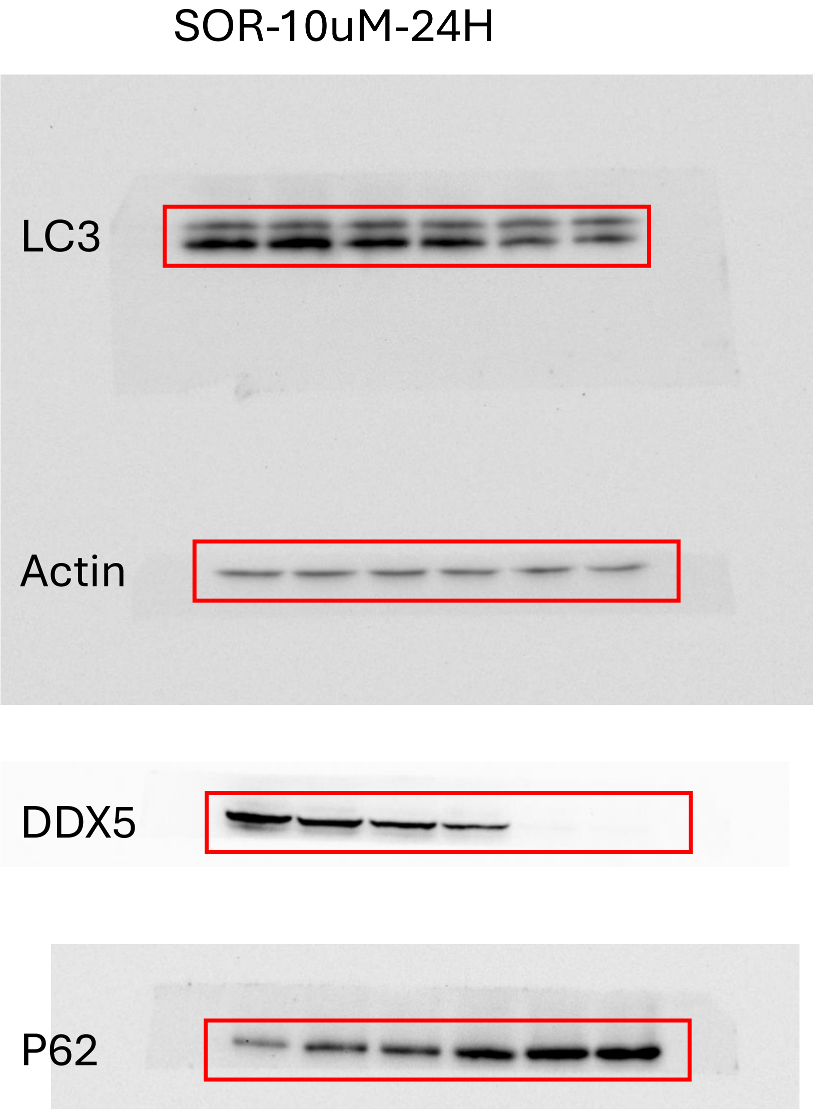

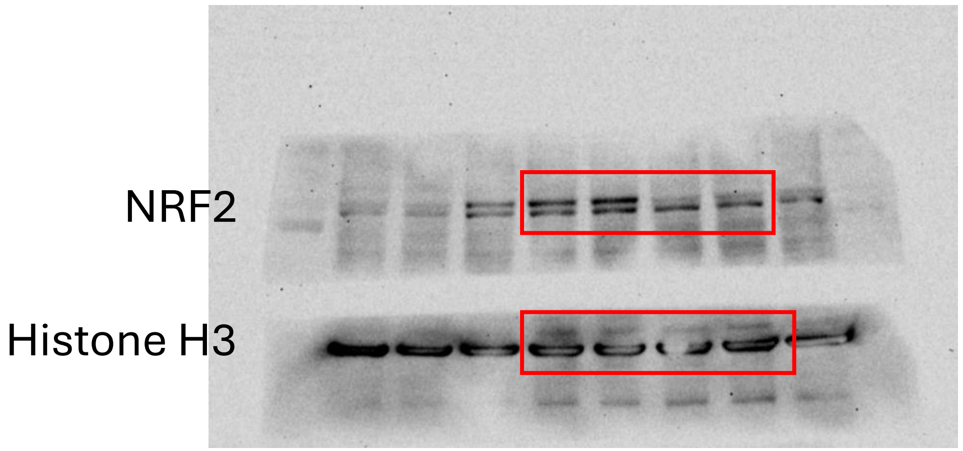
NRF2
Histone H3

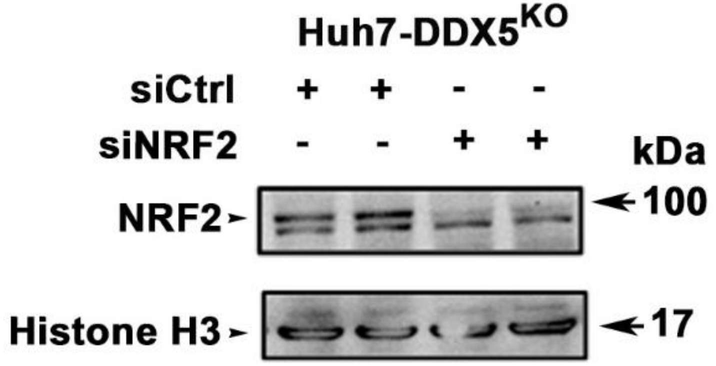
Huh7-DDX5KO
siCtrl + + - -
siNRF2 - - + +
kDa
NRF2
100
Histone H3
17
